# Biomimetic Polyurethanes in Tissue Engineering

**DOI:** 10.3390/biomimetics10030184

**Published:** 2025-03-17

**Authors:** Edyta Hebda, Krzysztof Pielichowski

**Affiliations:** Department of Chemistry and Technology of Polymers, Cracow University of Technology, Ul. Warszawska 24, 31-155 Kraków, Poland; krzysztof.pielichowski@pk.edu.pl

**Keywords:** polyurethane, non-isocyanate polyurethane, biomimetic materials, tissue engineering, soft tissue, hard tissue

## Abstract

Inspiration from nature is a promising tool for the design of new polymeric biomaterials, especially for frontier technological areas such as tissue engineering. In tissue engineering, polyurethane-based implants have gained considerable attention, as they are materials that can be designed to meet the requirements imposed by their final applications. The choice of their building blocks (which are used in the synthesis as macrodiols, diisocyanates, and chain extenders) can be implemented to obtain biomimetic structures that can mimic native tissue in terms of mechanical, morphological, and surface properties. In recent years, due to their excellent chemical stability, biocompatibility, and low cytotoxicity, polyurethanes have been widely used in biomedical applications. Biomimetic materials, with their inherent nature of mimicking natural materials, are possible thanks to recent advances in manufacturing technology. The aim of this review is to provide a critical overview of relevant promising studies on polyurethane scaffolds, including those based on non-isocyanate polyurethanes, for the regeneration of selected soft (cardiac muscle, blood vessels, skeletal muscle) and hard (bone tissue) tissues.

## 1. Introduction

The fascinating ability of living organisms to create complex structures has been the subject of many studies and scientific inquiries for years [1,2]. With increasing technical capabilities, we are able to imitate nature better and better. Biomimetics, understood as an interdisciplinary science that draws inspiration from nature when designing and producing new materials, goes a step further. The assumption of this field is not only an attempt to faithfully reproduce biological structures, but also to understand, describe, and use natural processes and optimal solutions. Biomimetic design is a breakthrough in the way of thinking about materials, because in principle, they are created for a specific target element, taking into account its desired features. Assuming that the structure affects the properties of the material, they can be matched to the given functions of the finished object. Nature’s inspiration is a promising tool for designing new biomaterials, especially for pioneering technological areas such as tissue engineering [3,4,5,6].

The term “biomaterial” was first defined during the Biomaterials Consensus Conference at the National Institutes of Health in 1982. In accordance with the findings, the following definition has been adopted since then: “Biomaterial is any substance (other than a drug) or combination of substances, synthetic or natural, that can be used at any time, and whose task is to supplement or replace the tissues of an organ or its parts in order to fulfill their functions” [7,8]. A common feature of all biomaterials is physiological acceptance by a living organism, which determines their use in medicine. It results from the similarity, in chemical, biological, and mechanical terms, of individual biomaterials to the tissues whose functions they are to replace or support. Very important properties characterizing biomaterials are the lack of toxicity and immunogenicity, i.e., their chemical and pharmacological inertness, so that they do not cause allergic, carcinogenic, or cytotoxic reactions. Depending on the purpose of the material, they can also exhibit such properties as bioactivity, degradability, biostability, osteoinductivity, and osteoconductivity [9,10,11,12].

## 2. Biomimetics in Tissue Engineering

The basis for the development of science dealing with the structure and regeneration of tissues was the discovery of the function of the extracellular matrix (ECM). It is a substance that creates a three-dimensional space, composed of collagen fibers, elastin, glycosaminoglycans, adhesive proteins, and water. It occurs throughout the body as a means of connecting cells in order to create specific tissues [13,14]. The polysaccharides contained in it, such as hyaluronic acid or heparin, have hydrophilic groups in their structure, responsible for binding water molecules, thanks to which the intercellular matrix takes the form of a gel. The basic tasks of the natural extracellular matrix include providing cells with appropriate conditions for proliferation. This is done by supplying oxygen and essential nutrients and removing unnecessary metabolic products from their environment. The key function of the ECM is to support and organize cells into structures through interactions between cell integrins and matrix fibronectins. It also provides protection against mechanical damage [15,16].

The natural extracellular matrix fulfills many important functions at the cellular, tissue, and organ levels. These functions and the properties of the ECM depend on the type of tissue. An important issue is to adapt the properties of the substrate to the properties of the regenerated tissue. The aim is for the synthetic extracellular matrix to exhibit biomimicry, i.e., to imitate certain beneficial features and fulfill some functions of the natural extracellular matrix [17,18,19].

Replacing this substance with a polymeric material and developing methods for culturing cells outside the human body allowed for the rapid development of this science and aroused the great interest of scientists in many fields, such as chemistry, biology, and tissue engineering [20,21]. Tissue engineering (TE) is a scientific discipline that proposes new, alternative solutions that change the approach to treatment with transplants and replacement of organ or tissue defects. The beginnings of tissue engineering date back to the 1980s. Later on, chemist J. Langer and surgeon J.P. Vacanti drew the attention of the scientific world to the potential and enormous possibilities of this young field [22]. The main goal of tissue engineering is considered to obtain a biological material that would make it possible to replace, restore, or maintain the basic functions of damaged tissues or entire organs. This material should have a biochemical structure that fully resembles natural tissue, with its extraordinary mechanical properties [23].

**Figure 1:**
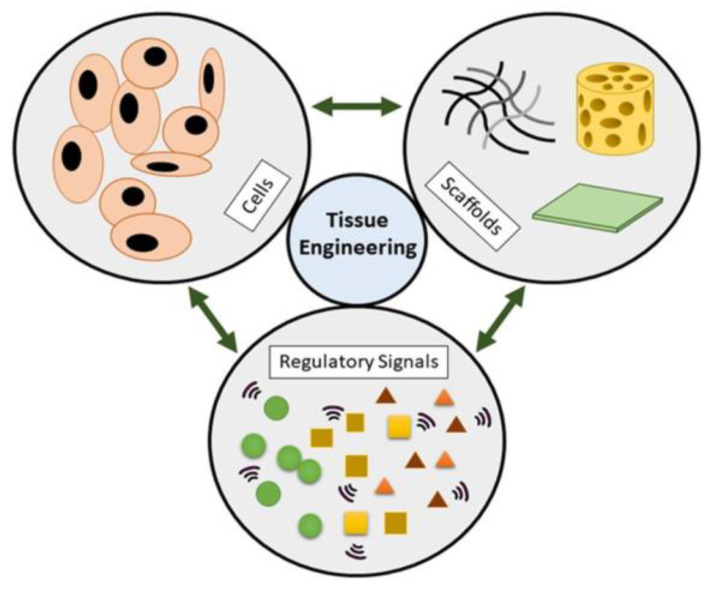
Triad of tissue engineering. Reprinted from ref. [24], Copyright (2021), MDPI.

The use of a combination of cells, scaffolds, and appropriate biomechanical factors in tissue engineering provides the possibility of improving or replacing biological functions [24]. These three key elements form the so-called triad of tissue engineering (Figure 1).

A cellular scaffold is a place of cell attachment, which provides them with specific spatial conditions necessary for proper growth. After being implanted on the scaffold, cells multiply and then integrate with the cells of the damaged tissue. Growth factors, i.e., vitamins, amino acids, sugars, or hormones, are nutrients that enable cells to function and grow properly [25]. Cell scaffolds serve as a temporary extracellular matrix (ECM), which provides support for cell growth and tissue regeneration [26,27].

Tissue substitutes prepared in vitro can be implanted in the place of a tissue or organ defect. Further regeneration takes place in vivo [28,29,30]. One of the most important parameters of tissue substrates is architecture—porosity, size, geometry, connection, and arrangement of pores in the material. High open porosity allows for the settlement and delivery of cells to the regeneration site, enables the transport of nutrients and metabolic products, as well as vascularization of the newly formed tissue. The hierarchical structure of pores considered at the level of milli-, micro-, and nanometers also plays a key role. The milli- level determines the shape and dimensions of the scaffold, as well as its mechanical properties. Microstructure affects cell migration and transport of nutrients and by-products. In turn, the nanometric structure ensures cell adhesion and also affects the expression of ECM components [18,30,31]. The optimal average pore size for newly formed vessels is 5 μm, for hepatocyte development about 20 μm, for skin regeneration 20–125 μm, and for bone tissue regeneration 100–350 μm. Pores with a diameter greater than 500 μm promote the formation of fibrous (scar) tissue and its rapid vascularization. The shape of the pores also affects the life processes of cells. It should allow both cell adhesion and flattening and for the cell to assume a spherical shape, necessary for carrying out specific life processes [32].

The mechanical properties of ECM, such as elastic modulus and density, have also a significant impact on cell differentiation, migration, growth, and proliferation [18,33,34]. Therefore, the best approach is to adapt the mechanical properties of the substrate to the properties of the natural extracellular matrix of a given tissue [17,35]. Mechanical stimuli transmitted to cells via a synthetic matrix have a positive effect on the secretion of components of the natural extracellular matrix and its reconstruction [18]. The second aspect of the mechanical properties of synthetic ECM is the creation of a temporary, structural support for cells. In many cases, there is also a need to temporarily take over the mechanical functions of a given tissue or organ [36]. The synthetic matrix allows for the transfer of some stresses and provides protection of cells against excessive loads until the developing tissue reaches sufficient strength [37,38]. The tissue scaffold should also maintain dimensional and structural stability after implantation, until the tissue is developed. This is particularly important in the case of hard tissues—bone or cartilage tissue [32].

The desired architecture and mechanical parameters of the scaffold can be obtained primarily by selecting appropriate methods of obtaining them, and using various materials, including (bio)composites. The key challenge is to ensure the appropriate porosity and mechanical properties of the substrate, enabling simultaneous migration and communication of cells, transport of nutrients and by-products, providing the environment necessary for cell differentiation and proliferation, and transferring some of the stresses.

In addition to the synthesis stage of an appropriately designed cell scaffold, the process of reconstructing new tissue also includes the element of cell culturing on the obtained substrate. There are two methods of applying cells to a synthetic matrix. The first one involves applying cells, which can be collected from both the donor and the patient, outside the human body. However, autologous cells do not require subsequent treatment with immunosuppressive drugs. In the second method, the scaffold without cells is implanted in the place requiring repair, where only after some time will the cells spontaneously settle on the substrate [39].

**Figure 2:**
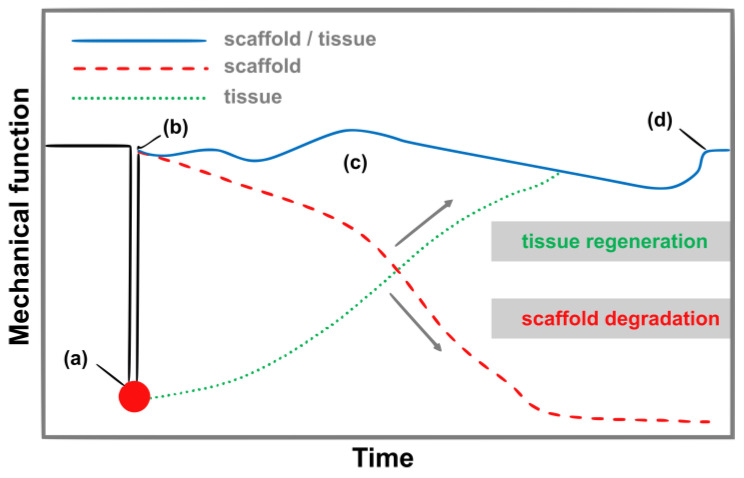
Illustrating the changes in the mechanical function of a biodegradable scaffold based on regenerated bone tissue: (a) tissue loss; (b) tissue scaffold implantation; (c) tissue regeneration (scaffold degradation); (d) complete tissue reconstruction.

The cultivation of cells on the obtained scaffold in the in vitro environment begins with the collection of donor cells. There are three types of cells: autologous, taken directly from the patient; allogeneic, which come from a donor who is another person; and syngeneic, which come from a genetically identical individual. Depending on the type of tissue being recreated, the types of cells that have the ability to rebuild it are collected. The smallest elements of living matter that can revolutionize tissue engineering are so-called stem cells that can differentiate without restrictions into various types of cell lines, depending on the type of growth factors used. They can be isolated from bone marrow or even from adipose tissue collected during liposuction [40]. The isolated cells are multiplied in the in vitro environment and then transferred to a sterilized scaffold. An important element is the selection of appropriate growth factors, which are also applied to the substrate. The scaffold supplied with a medium and the multiplied cells are placed in a bioreactor in the next stage. In this device, conditions similar to those prevailing in the in vivo environment are ensured, i.e., appropriate temperature, humidity, oxygenation, and pH. After a specified period of time, when the degree of cell multiplication is sufficient, such a scaffold is implanted into the human body. The cells present on the scaffold have the ability to produce and excrete collagen into the intercellular space, which is then organized into a natural extracellular matrix [41,42,43]. Over time, as a result of metabolic changes, the tissue scaffold biodegrades into non-toxic products that are excreted from the body. It is important that the degradation of the material occurs in a controlled manner (Figure 2). The time of decomposition of the biomaterial must be matched to the time of tissue regeneration [44,45].

Too rapid degradation may not only cause premature loss of mechanical properties, but also the release of a significant amount of degradation product in a short time, exceeding the body’s ability to excrete it. The direct effect of this is then prolonged inflammation. This is particularly important in the case of polymers whose transient degradation products are acidic (e.g., polylactides) [46,47]. On the other hand, the degradation of the material should not be too slow. In optimal conditions, the polymer implant should undergo gradual resorption, proceeding in accordance with the ongoing tissue healing process [48,49,50].

There are numerous examples of polymers used in the production of scaffolds in the literature. All collected polymers can be categorized into four types of polymers due to the source of raw materials and the method of synthesis:

- Natural origin;
- Bacterial origin;
- Synthetic;
- Synthetic from renewable raw materials [51].

The first polymers considered in the process of scaffold synthesis were natural proteins and polysaccharides. This choice was motivated by their bioactive properties, which have an impact on better cell adhesion to the substrate. However, it quickly turned out that their use is also associated with the possibility of the occurrence of malignant pathogens, high cost of acquisition, and limited way of modifying their properties and biodegradation parameters. In response to the problems related to the use of natural polymers, replacing them with synthetic macromolecular compounds has undoubtedly been successful. Synthetic polymers are extremely useful due to highly developed control of their properties during the synthesis process. Through the conditions of the reaction, it is possible to influence mechanical strength, physicochemical properties, and degradation time, and in this way obtain materials adapted to specific needs [52].

An additional advantage of producing synthetic biomaterials is the control of the chemical composition and thus the impurities present in it. In this group of polymers, biodegradable polymers, which are currently the subject of intensive research, should be distinguished [50].

The most popular representatives of this group are aliphatic polyesters, which are widely studied and used in tissue reconstruction processes. However, apart from them, there is a very large group of biodegradable polymers that can also be successfully used. These include hydrophilic polymers, such as poly(ethylene glycol), or hydrophobic polymers that are resistant to mechanical stress and the action of hydrolytic enzymes, e.g., poly(ethylene terephthalate), poly(alkyl siloxanes), fluorinated polymers, and polyurethanes [53].

The last group of polymers—polyurethanes (PUs)—are often used in biomedical applications due to their good flexural and tensile strength and good biocompatibility. Moreover, it has been shown that the absorption of plasma proteins on polyurethanes is slower or smaller than on other materials [54,55]. Therefore, polyurethanes have become a good material for various medical applications requiring unique antithrombotic and biomimetic properties. Additionally, obtaining biodegradable polyurethanes has significantly increased the possibilities of their applications, for example, in tissue engineering [53].

## 3. Polyurethane Chemistry

**Figure 3:**
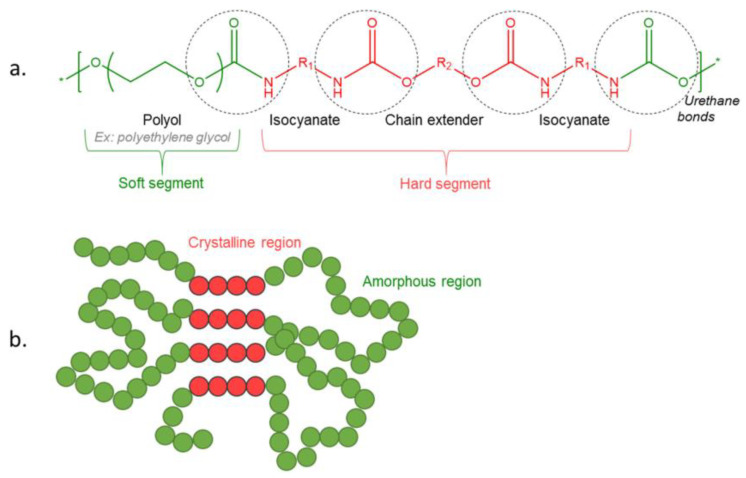
(**a**) Schematic representation of a semi-crystalline polymer and (**b**) linear polyurethane structure. Reprinted from ref. [60], Copyright 2022, Elsevier.

The use of polyurethanes in biomedicine is mainly related to their unique multiphase system morphology, which plays a fundamental role in the final properties of polymers, yielding, for example, a variety of mechanical properties. The molecular architecture of polyurethanes can be controlled and modified by changing the chemical nature of the substrates and the processing method. Therefore, precise determination of the morphology is necessary to understand the relationship between the PU structure and properties. The morphology of polyurethanes is complicated due to the phase separation of crystalline and amorphous microdomains, which form rigid and flexible segments, respectively. Additionally, phenomena such as crystallization and hydrogen bonding may also strongly affect the polymer morphology. The degree of phase separation depends on the molar mass of the substrates, their chemical nature, and the mutual interaction between the flexible and rigid segments. For example, a greater degree of phase separation is seen in polybutadiene-based polyurethanes than in polyethers and polyesters [56,57,58,59]. Figure 3 shows semi-crystalline polymer and PU linear structures, as well as PU foam morphology [60].

The structure of a polyurethane is determined by the distribution of rigid and flexible segments, as well as their size and degree of cross-linking. These structures are described in terms of chain conformation and affect the degree of crystallinity phase separation. Both primary and secondary structures affect the final properties of the polymer [57,59,61]. The morphology is also influenced by aspects such as phase volume, size, shape, orientation, and the interconnection of comonomers, depending on the percentage of rigid segments and thermal history [61].

In order to create a material with elastic properties, the melting temperature of the crystalline phase must be higher than the operating temperature. On the other hand, the elastic segment, which is mainly composed of amorphous domains, should be only slightly crystalline, and its glass transition temperature must be significantly below the operating temperature. Only when these two conditions are met do the crystalline domains function as a network node, and the amorphous phase freely binds them together [62]⁠. It follows that the elastic segment affects the lower range of the operating temperature and the elasticity of the material, while the rigid segment determines its upper range and the modulus of elasticity, hardness, and strength [63,64]⁠. Therefore, the structure of the polyurethane elastomer has a fundamental influence on the final properties of the product.

The phase separation itself is determined by several factors. One of them is the geometrical conditions of the rigid segment and its ease of mutual adjustment and formation of crystalline rigid domains of polar character, separating from apolar macrodiol segments forming amorphous domains. The rigid segment consists of isocyanate and chain extender, so these two components will affect its geometry [65]. Also important are the structural features of the polyol, isocyanate, and chain extender molecules, such as chain length, chemical structure, and functionality, which have a significant impact on the properties of polyurethane. All these parameters can affect the way rigid segments are arranged in the crystalline domain and the degree of phase separation. The physical properties of PUs are dependent on the cross-linking density and intermolecular forces between polymer chains. Even when a cross-linking agent is not used, cross-linking can occur during synthesis through the formation of allophanate and biuret groups.

Intermolecular hydrogen bonds have a high impact on the phase separation. The hydrogen bond energy is at the level of 20–50 kJ/mol, making it the strongest of the secondary interactions [59]. In the case of polyurethanes, the degree of bonding by these bonds increases in the following order due to the type of polyol used: polybutadiene < polyether < polyester. Hydrogen bonds in polyurethanes occur primarily between the carboxyl group and the secondary amine group, which occur in the urethane bond. The bonds formed in this way cause the extension of the bond between the nitrogen and hydrogen atoms in amines, as well as between the carbon and oxygen atoms in carbonyls. Such extension causes a decrease in the frequency of bond vibrations, which is visible as a separation of the absorption band under infrared irradiation. Thanks to this, hydrogen bonds can be detected by infrared spectroscopy; free and hydrogen-bonded bands of carboxyl and amino groups are studied in particular [66,67,68,69,70]. By comparing the intensity of the bands free from hydrogen bonding and the bond participating in the formation of hydrogen bonding, it is possible to determine with some approximation the degree of phase separation. Hydrogen bonding plays a fundamental role in phase separation. At room temperature, about 90% of amino groups in rigid segments are hydrogen-bonded [67,71,72], while with increasing temperature, hydrogen bonds disappear as the glass transition temperature of rigid segments is approached. Despite this, 30–40% of hydrogen bonds are maintained even up to 200 °C [67].

Designing biodegradable polyurethanes (PUs) requires taking into account many complex factors that affect their properties. The ratio of components, such as the amount of diisocyanate and polyol, is crucial because it can significantly affect the hardness, elasticity, and biodegradability of the material. The mechanical properties of PUs, such as tensile strength, flexural strength, and elongation at break, are closely related to their microstructure. For example, the presence of hydrogen bonds can improve structural stability, and appropriate phase separation can affect elasticity and resistance to external factors. In addition, changes in molecular weight can affect the rate of degradation of the material, which is particularly important in the context of biomedical applications, where the degradation time of PUs must be tailored to specific needs, such as wound healing time or drug release.

The complexity of this subject opens up many opportunities for innovative applications, such as materials for the production of implants, as well as materials for tissue regeneration. By adjusting the chemical composition and parameters of the production processes, it is possible to obtain PU with specific properties, which makes it an ideal solution in various fields of medicine and biomaterials technology.

## 4. Soft Tissues, Hard Tissue

**Figure 4:**
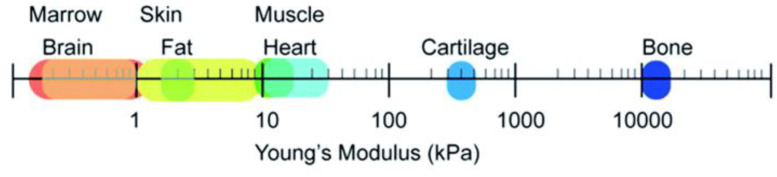
Tissue elastic modulus as a reference for the design of suitable scaffolds. Reprinted from ref. [78], Copyright 2022, Royal Society of Chemistry.

PU elastomers are rubber-like materials with a transition temperature lower than room temperature that exhibits biocompatibility, fatigue resistance, and durability. PU generates potentially toxic by-products and is considered a non-degradable material. However, by integrating unstable hydrolyzable groups into the PU backbone, an elastomeric biodegradable PU can be created. The biodegradability can be introduced into PUs by using degradable polymers as soft and/or hard segments, such as polycaprolactone (PCL) [73,74], poly-l-lactide (PLA) [75], poly(glycolic acid) (PGA) [75], and their copolymers [76]. The presence of terminal hydroxyl groups enables the synthesis of alternating segments in the PU chain. Hard segments in PU increase the hardness, tensile strength, impact resistance, stiffness, and modulus of the material, while soft segments increase water absorption, elongation, flexibility, softness, and degradability. Due to the possibility of the tunability of physicochemical properties, biodegradable PUs are attractive substrates in regenerative medicine. For example, the composition of PUs can be appropriately matched to the function of the tissue that requires replacement. Due to differences in the mechanical properties of tissues (Figure 4), rigid polyurethane scaffolds are suitable for hard tissue replacement, while soft polyurethane structures better meet the requirements of soft tissue [77].

Elastomeric biodegradable polyurethanes are promising candidates for the regeneration of various soft tissues, including cardiac muscle [79], blood vessels [80,81], skeletal muscle [82], and cartilage [83], and the repair of tendons, ligaments, and skin. This class of materials is also being investigated for hard tissue regeneration, such as bone tissue repair [53].

## 5. Hard-Tissue Engineering

### 5.1. Bone Tissue Engineering

Bone grafting is often needed to accelerate the healing of bone defects. In addition to autografting, a standard procedure involving bone taken from the patient’s own body (often from the iliac crest), it is possible to use a biodegradable or bioresorbable synthetic material with similar mechanical properties to bone. In particular, tissue engineering approaches to repair or regenerate bone tissue function include the combined use of osteoconductive biodegradable scaffolds, osteogenic cells, and osteoinductive bioactive factors. Signaling molecules such as growth factors can be incorporated into the scaffold to enhance its osteoconductive properties. In terms of mechanical properties, scaffold materials should optimally match the properties of the tissue to be replaced [84,85].

Bone tissue engineering is a rapidly developing field that focuses on creating new methods for treating bone-related diseases. Unlike traditional approaches, such as allografts (grafts from other people), autografts (grafts from own body), and xenografts (grafts from other species), bone tissue engineering seeks to find alternatives that could minimize the risk of rejection, infection, and other complications [85].

The design of biomimetic bone material aims to create artificial bone grafts that are superior to autologous and allogeneic grafts and provide full regeneration of bone defects. Based on the definition of biomimetic bone scaffold, Xu et al. [86] proposed that the design and fabrication process of these scaffolds should be based on four key principles:

(1) Compositional biomimetics: materials should mimic the natural chemical composition of bone, which can affect their biocompatibility and ability to integrate with surrounding tissues.
(2) Structural biomimetics: the structure of the scaffold should mimic the hierarchical organization of bone, which can improve the mechanical properties and functionality of the grafts.
(3) Mechanical strength biomimetics: materials should have adequate strength and flexibility to meet the biomechanical demands of the body.
(4) Biomimetic regeneration process: the design should take into account the natural processes of bone healing and regeneration, supporting cell proliferation and new bone formation.

**Figure 5:**
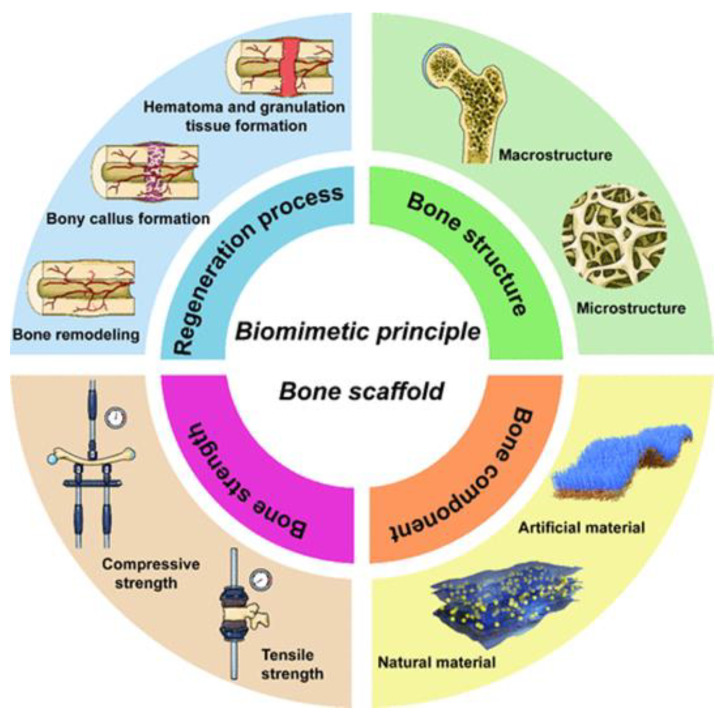
New biomimetic principles for bone scaffold fabrication. Reproduced with permission from ref. [86], Copyright 2024, ACS Publications.

Fulfilling these four principles (Figure 5) aims to create biomimetic bone materials that will be a more effective substitute for autologous bone, thus improving clinical outcomes and the quality of life for patients.

The bone tissue engineering field encompasses a wide range of medical procedures, including orthopedic defects, bone tumors, spinal segment stabilization, pseudoarthrosis, and various interventions in maxillofacial, orthopedic, reconstructive, and trauma surgery [86].

Bone is a living, dynamic tissue that is constantly remodeling. Studies have shown that 10–15% of the bone in the body is replaced with new bone each year [87]. Bone matrix is a three-dimensional structure secreted by cells into the extracellular space. It is composed of organic (40%) and inorganic (60%) compounds, and the exact composition varies with gender, age, and health status. The main inorganic components of ECM are calcium-poor apatite and trace elements. On the other hand, organic ECM is much more complex, consisting mainly of type I collagen (90%) and non-collagen proteins (10%). It is mainly synthesized by osteoblasts before the mineralization process [87].

The bone ECM dynamically interacts with osteoblasts and osteoclasts, regulating the processes of bone remodeling and regeneration. The bone remodeling process occurs thanks to specialized cells of bone tissue, among which we can distinguish [88]:

- Osteoblasts, which are responsible for bone synthesis;
- Osteoclasts, which are responsible for bone resorption;
- Osteocytes and lining cells, which are permanently present in bone tissue.

Due to their structure, location, and function, compact and spongy bone are distinguished. One difference between these two types is their porosity. The apparent density of solid compact bone is about 1.8 g/cm^3^, while the relative density, i.e., the ratio of the apparent density of the sample to the mass of solid bone, reaches a value of >0.7. The mechanical properties of spongy bone, which consists of many trabecular elements with dimensions greater than 1 mm, are called apparent mechanical properties. These properties depend on the characteristics of the bone tissue matrix, the amount of tissue, and the spatial organization of the trabeculae. The relative density and architecture of spongy bone are of great importance. Since the relative density and architecture of cancellous bone vary depending on the anatomical site, age, and past or permanently acquired diseases, there are significant differences in bone stiffness and strength [89].

Depending on the extent of the defect, we can distinguish three methods of bone regeneration: (1) synthetic scaffolds, (2) scaffolds combined with active particles, and (3) materials obtained in combination with cells. Tissue engineering allows an alternative approach to the creation of bone grafts by providing temporary, artificial scaffolds for the growth and regeneration of natural tissue [90]. In relation to bone tissue, tissue engineering methods allow the production of a three-dimensional (3D) structure with controlled porosity in order to create an environment supporting bone growth. It is desirable to obtain bone scaffolds from biodegradable materials, which is why they are completely replaced by new tissue [91]. A bone scaffold is a mechanical structure designed to mimic the extracellular matrix (ECM) of bone tissue, which creates conditions for bone remodeling processes to occur with minimal complications [92]. Implantation of bone scaffolds is successful if mesenchymal stem cells (MSCs) contact and adhere to the material surface [93]. After the initial stage, bone begins to grow on the outer surfaces of the scaffold with an internal mineralization gradient [94].

The success of this process depends on several critical parameters of the scaffold, such as surface roughness, internal architecture, porosity, the chemical composition of the material, and biocompatibility [95]. Bone scaffolds in tissue engineering can be made of various types of materials, such as metals, ceramics, and polymers. Metal scaffolds are used due to their osteoinductive properties, but they are not biodegradable. Ceramic scaffolds exhibit biomimetic properties, but are stiff and have limited biodegradability and limited processability. Polymer scaffolds are characterized by good processability, a controlled degradation rate, as well as the ability to adjust mechanical properties and wettability to the conditions of their use, which makes them the appropriate candidates in bone tissue regeneration [96]. To achieve the optimal biomimetic effect, it is advisable to combine the benefits of several materials in accordance with biomimetic therapy principles.

#### Polyurethane Scaffolds for Bone Engineering

Biodegradable polyurethane elastomers, thanks to their tunable mechanical properties, are excellent materials for bone-tissue engineering. In order to improve the osteogenicity of biodegradable polyurethane scaffolds, they are combined with an osteogenic material. An example of this type of modification is the work of McEnery et al. [97], who synthesized a new thioketal (TK) diol and then cross-linked it with a lysine triisocyanate (LTI) prepolymer. The obtained poly(thioketal urethanes) (PTKURs) are hydrolytically stable but are subject to degradation by reactive oxygen species (ROS) released by cells. They were then combined with MasterGraft™ (MG; Medtronic, Memphis, TN) ceramic blend (85% β-tricalcium phosphate/15% hydroxyapatite) to produce PTKUR/ceramic composite cements that, when implanted into femoral condyle defects in rabbits, supported appositional new bone growth, osteoclast-mediated resorption, and host bone integration observed at 6 and 12 weeks after implantation. The cements also demonstrated initial compressive strengths exceeding that of trabecular bone, and working times comparable to commercial bone cements (5–10 min).

McGough et al. [98] investigated PTKUR/ceramic composites as iliac crest (AG) autograft extenders. PTKUR/AG and PTKUR/CaP/AG showed operating times of 10–20 min and compressive strengths of 1–2 MPa. When implanted in a rabbit posterolateral intertransverse process bone formation model, bone growth along the posterior plane of the processes was consistent from 2 to 8 weeks for PTKUR/AG and PTKUR/CaP/AG. New bone formation was also evident in the intertransverse process (ITP) space away from the processes. Incorporation of PTKUR/CaP/AG showed that the AG content could be reduced to approximately 50% without significantly impairing bone healing. This work provides evidence for the high potential of PTKUR-based AG extensions in the treatment of bone defects.

To enhance the colloidal stability, handling properties, and osteoinduction of nanocomposites, the same research group [99,100] introduced hydroxyapatite nanocrystals (nHA-PTKURs) into PTKUR, which were implanted into femoral defects in white rabbits to assess ossification at 4, 12, and 18 months of age. The results suggest that nHA-PTKUR cements support combined intramembranous and endochondral ossification, which results in enhanced cement osseointegration and potentially may improve patient outcomes.

Talley et al. [101,102] synthesized low-viscosity polyester polyurethane (LV-PUR) and then obtained the composites by doping with 85% β-tricalcium phosphate/15% hydroxyapatite (CM) or bioactive glass (BG). They also incorporated recombinant human bone morphogenetic protein-2 growth factor (rhBMP-2), which promotes the differentiation, maturation, and proliferation of multivascular cells. Injectable bone grafts were placed in a rabbit posterolateral fusion (PLF) model. μCT images and histological sections revealed evidence of bone union. This study highlights the potential of rhBMP-2-loaded LV grafts as injectable bone grafts for MIS posterolateral spinal fusion surgery.

Studies by Lavrador et al. [103] have shown that biodegradable polyurethane (PU) foam with synthesized polycaprolactone (PCL) and HMDI segments, combined with a 1,4:3,6-dianhydro-d-sorbitol chain extender, has potential in medical applications, especially in tissue engineering. Scaffolds made of this foam, enriched with nano-hydroxyapatite (nHA) and bioactive compounds (such as creatine/putrescine or soy lecithin), were implanted into defects in the tibial and iliac crest of sheep. After 26 weeks of observation, new bone growth was observed both around and inside the PU scaffolds. These results emphasize the importance of the porosity of these scaffolds, which allows cell infiltration and promotes bone tissue regeneration processes. This study suggests that appropriately designed biomimetic materials may support bone regeneration and be useful in the treatment of bone defects.

Lei et al. [104] in their study focused on the development of a porous polyurethane adhesive (PUA) and the evaluation of the influence of different components, such as water, polyisocyanate, and β-tricalcium phosphate (β-TCP), on the physicochemical and mechanical properties of this material. By modifying the water content, PUA with a different surface morphology and porosity was obtained, which had a direct impact on its properties. The addition of polyisocyanate and β-TCP proved effective in increasing both the adhesive strength and mechanical properties of the adhesives. Importantly, the adhesion strength of PUA to bone was twice that of clinical bone cement based on poly(methyl methacrylate), suggesting its potential advantage in medical applications. In vitro analyses, including cell culture and adhesion tests, showed the high biocompatibility of PUA. In addition, in vivo studies in a rabbit model showed that the porous structure of the adhesive promotes cell growth and bone tissue regeneration. PUA also showed a compressive strength and modulus that were similar to those of cancellous bone, which is an important advantage in the context of its use as a bone gluing material. All these results indicate that the porous polyurethane adhesive has the potential to become an effective solution for bone regeneration and reconstruction through bone gluing.

In the work of Zhu et al. [105], the authors investigated the ability of TiO_2_ nanoparticles to improve the mechanical properties of scaffolds, promote biomineralization, and support the proliferation of osteogenic cells. Since unmodified TiO_2_ nanoparticles cannot distribute evenly in fibers and do not show a reinforcing effect, the poly(ester urethane)urea (PEUU) scaffold was first reinforced by grafting TiO_2_ onto poly(ester urethane) (PEU) to obtain PEU-g-TiO_2_. The scaffolds were fabricated by electrospinning to obtain three kinds of fibers: pure PEUU scaffold, PEUU scaffold with unmodified TiO_2_ nanoparticles (TiO_2_/PEUU), and PEU-g-TiO_2_/PEUU. PEU-g-TiO_2_/PEUU significantly increased the Young’s modulus and tensile stress of PEUU scaffolds, while unmodified TiO_2_ significantly decreased the Young’s modulus and tensile stress. The greatest strengthening effect was observed for the 1:1 ratio PEUU and TiO_2_-modified PEU scaffolds. PEU-g-TiO_2_/PEUU scaffolds also showed a higher biomineralization capacity than PEUU scaffolds. Moreover, these scaffolds promoted MSC growth better than pure PEUU scaffold and TiO_2_/PEUU scaffold. The obtained scaffolds, alone or in combination with osteogenic cells, have the potential to regenerate bone.

**Figure 6:**
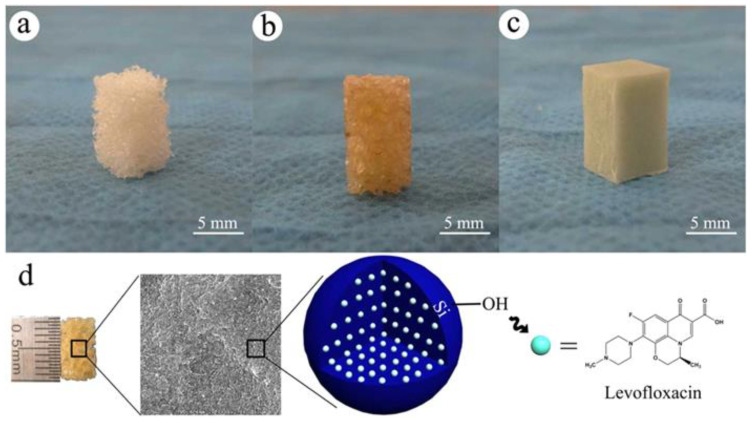
The shape and internal structure of new synthetic composite scaffolds. The HA/PU composite porous scaffolds were manufactured using the situ foaming method. The size of the material was 10 mm × 6 mm × 6 mm (**a**). Lev/MSNs/n-HA/PU scaffolds were combined with MSNs, which contained different concentrations of levofloxacin (**b**). PMMA cement that contained 1 mg or 5 mg levofloxacin (**c**) was used as a positive control group. Levofloxacin was successfully loaded with mesoporous silica nanoparticle via electrostatic attraction (**d**). Reproduced with permission from ref. [106], Copyright 2017, Springer Nature.

Synthesized by Huang et al. [106], a novel levofloxacin-loaded mesoporous silica microspheres (MSNs)/nano-hydroxyapatite/polyurethane composite (Lev/MSNs/n-HA/PU) scaffold represents an innovative approach to the treatment of bone defects (Figure 6). As a result of this material’s composition, a degradable scaffold was formed that not only releases drugs but also exhibits bioactivity, which promotes bone tissue regeneration. The therapeutic effect in the treatment of chronic osteomyelitis with bone defects was evaluated in a rabbit model compared to bulk PMMA. The degradation of this composite starts after 12 weeks from implantation, which ensures that, until then, the material maintains its structural integrity, offering adequate mechanical support for the bone healing process. After the degradation of the material, new bone tissue is formed. This novel antibiotic-loaded biocomposite scaffold may serve as a drug delivery system for treating bone defects due to chronic osteomyelitis. However, as indicated, the use of antibiotics, including levofloxacin, may lead to the development of bacterial resistance.

Therefore, the next goal of the Huang [107] team was to investigate the osteogenic activity and antibacterial capacity of Lev/MSNs/n-HA/PU material in vitro. They found that both Lev/MSNs/n-HA/PU and n-HA/PU had the ability to inhibit the growth of bacterial colonies, but Lev/MSNs/n-HA/PU material showed stronger antibacterial activity and lower bacterial adhesion compared to n-HA/PU. The results of the study suggest that the Lev/MSNs/n-HA/PU composite scaffold provides favorable compatibility in vitro, which leads to the induction of osteogenic differentiation of stem cells (MSCs), promotes the proliferation and differentiation of MC3T3-E1 cells, and inhibits apoptosis. Additionally, a significant antibacterial effect of this material was observed in vitro. These results indicate the high potential of the Lev/MSNs/n-HA/PU composite scaffold as a material with a dual function: anti-infective and supporting osteogenesis, which may have important implications for future clinical applications.

Lin et al. [108] also studied bone scaffolds composed of polyurethane scaffold filled with nanohydroxyapatite (n-HA) and ciprofloxacin hydrochloride (CF), which is an antibiotic commonly used in the treatment of osteomyelitis. This study confirmed that CF scaffolds showed antibacterial properties, while the addition of n-HA provided the scaffolds with enhanced osteogenic differentiation.

**Figure 7:**
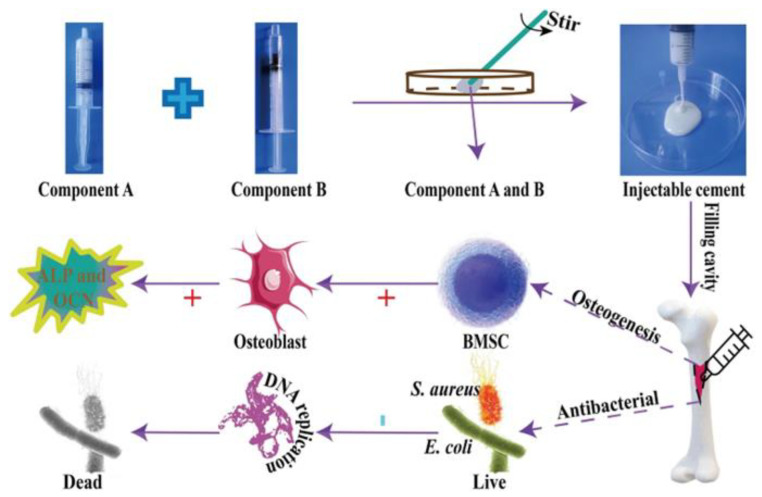
Injectable EN-loaded bone cement based on PUHA promoting osteogenesis and inhibiting bacterial. Reproduced with permission from ref. [111], Copyright 2022, Springer Nature.

The use of drugs/antibiotics alone leads to the increase in pathogen resistance; therefore, Huang et al. [109] designed another nano-hydroxyapatite/PU (Ag/n-HA/PU) composite containing 3% silver. Silver ions are known for their antibacterial properties and can effectively combat the bacteria responsible for osteomyelitis and infections associated with bone gaps. Good bone defect repair, a reasonable degradation rate of scaffolds, and no significant toxicity were observed, indicating the advantages of this new synthetic scaffold as a potential treatment option for chronic osteomyelitis. In general, antibacterial activity in the biomedical perspective is associated with compounds or elements that locally retard bacterial growth or annihilate bacteria without being generally toxic to the surrounding tissues. Most of the current antibacterial agents available in the medical industry are a chemical modification of natural compounds [110]. An example of an innovative approach is the work of Zhang et al. [111], who developed an injectable antibacterial bone cement by combining Enoxacin (EN) with a polyurethane/nano-hydroxyapatite (PUHA) composite—Figure 7.

Enoxacin, a third-generation fluoroquinolone antibiotic, has a broad spectrum of activity, especially against Gram-positive bacteria [112]. Its minimum inhibitory concentration (MIC) is much lower compared to the used inorganic antibacterial agents, such as silver or gold [113]. For this reason, EN is a suitable choice in the context of biomaterial-associated infections (BAIs). The antibacterial cements showed good viscosity and injectability, with shear behavior ensuring convenient manipulation. Adding EN drug accelerated the conversion of monomers and increased the cross-linking and mechanical properties, which enhanced the stability of the cured EN-loaded cements, which is beneficial to avoid the inflammatory reaction caused by the residual monomers. All the drug-loaded PUHA cements showed antibacterial properties.

Luo et al. [114] obtained 3D programmable polyurethane scaffolds based on polytetrahydrofuran and hexamethylene diisocyanate (HDI), in which excess HDI was gas-foamed with water to form interconnected pores. Citrate-functionalized calcium phosphate (CACP) was introduced into the scaffolds, enhancing the expression of osteogenesis and causing antibacterial activity. The obtained PTHF/CACP implant scaffolds were dedicated to repair the bone of a tooth defect. These scaffolds have a porous structure resembling bone and appropriate mechanical strength, which makes them suitable for supporting cells, which is crucial in tissue regeneration processes. The shape memory effect that these scaffolds achieve at 37 °C indicates their potential in applications where minimal invasiveness is important. Thanks to this, they can be used in various medical procedures that require precise adjustment and stability in the patient’s body. In addition, the increasing antibacterial coefficient for Escherichia coli and Staphylococcus aureus with increasing CACP content suggests that these scaffolds may also play a role in preventing infections, which is important in the context of implants. The high biocompatibility confirmed in vitro and osteoconductive capacity of cells indicate that PTHF/CACP scaffolds may be safe for use in living organisms. This makes them promising materials for further research and development in the field of regenerative medicine.

The work of Rahmani-Moghadam et al. [115] describes the preparation of a new biomaterial that combines hydroxyapatite (HA) with polyurethane (PU) in the form of a foam. The production process of HA-PU foam involved casting the polyurethane foam and then sintering it at high temperatures (800 and 1250 °C). The obtained HA-PU scaffolds were then impregnated in alginate (AL) hydrogels loaded with thymoquinone (TQ), which was applied to accelerate the bone regeneration processes. Thymoquinone was chosen due to its properties that can induce bone morphogenetic protein 2 (BMP-2) formation, which is crucial for the differentiation of cells into osteoblasts. The results of the study suggest that impregnation of HA-PU scaffolds in alginate hydrogels loaded with TQ leads to a significant acceleration of the differentiation process of human adipose tissue-derived mesenchymal stem cells (ADMSCs) into osteoblasts. An increase in the expression of osteoblastic markers, such as collagen, osteopontin, osteocalcin, and alkaline phosphatase, was observed, which is a positive indicator of cell differentiation. Additionally, the studies showed a slower degradation rate and an increase in the mechanical strength of the material, which suggests a synergistic effect of hydroxyapatite and thymoquinone on MSC differentiation. However, it should be noted that the authors did not conduct in vivo studies, which limits the possibility of fully assessing the efficacy and safety of the developed biomaterial.

Mesenchymal stem cells (MSCs) from umbilical cord blood, also known as UCB MSCs, are becoming increasingly popular in the context of regenerative medicine, especially in the regeneration of skeletal tissues. Their unique properties, such as the ability to trilineage differentiate into bone, cartilage, and fat cells, make them a promising alternative to adult stem cells. Compared to MSCs derived from adult tissue, UCB MSCs show a higher proliferation capacity and more favorable immunomodulatory properties. This means that they have the potential to modify the body’s immune response, which may be crucial in the context of cell therapy and transplantation [116]. In addition, the growing interest in cord blood banking makes MSCs from this source easily accessible for clinical applications. This availability, combined with their regenerative properties, makes them an attractive option for tissue engineering, especially in the context of treating bone and cartilage defects. Therefore, the work of Tahlawi et al. [117] describes an attempt to functionalize the peptide arginyl-glycyl-aspartic acid (RGD) on the surface of porous polyurethane scaffolds (PU-RGDs) to be used for the expansion and osteogenic differentiation of umbilical cord blood mesenchymal stem cells (UCB MSCs). The results of the study suggest that umbilical cord blood-derived stem cells (UCB MSCs) show a uniform increase in cell adhesion and number in different regions of the scaffold, which may be important for the efficacy of engineered bone grafts. Increased osteogenic differentiation of these cells may contribute to better bone tissue production, which is crucial in the context of clinical applications. Such results may open new possibilities in the treatment of bone injuries and in bone tissue regeneration, offering potentially more effective and safe therapeutic methods.

**Figure 8:**
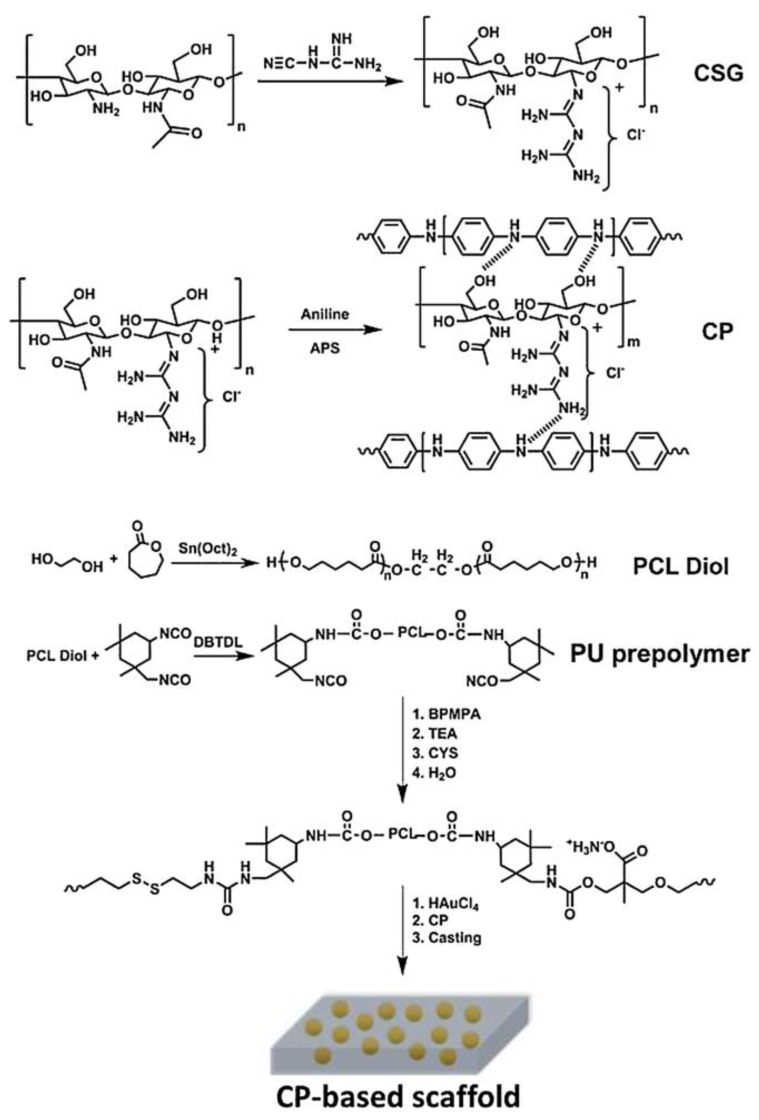
The schematic presentation of the preparation route of CP-contained scaffolds. Reproduced with permission from ref. [118], Copyright 2021, Elsevier.

Shaabani et al. [118] obtained advanced hybrid scaffolds developed using chitosan biguanide (CSG) and polyaniline (PANI), which possess self-healing and electrically conductive properties. These scaffolds, obtained by polymerization of aniline in the presence of CSG and polyurethane (PU) prepolymer (Figure 8), combine various advantageous features that make them promising for bone tissue engineering. CSG provides hydrophilicity and hydrogen interactions, which affect biological properties, while PANI contributes to high conductivity and the possibility of tunable electrical properties of the scaffold. PU, in turn, provides strong mechanical properties and biocompatibility, whereby its degradation products are non-toxic.

The results of the study showed that the prepared scaffolds show an excellent shape memory effect and self-healing ability at a temperature close to body temperature. An important aspect is also that they promote HAMSC cell proliferation and matrix mineralization, increasing the expression of genes related to bone tissue reconstruction, such as COL-1, ALP, RUN2, and OCN. The authors indicate that this type of scaffold has great potential in repairing cancellous bone defects, but the complicated design of the scaffolds may be an obstacle to their implementation in clinical trials.

Biomimetic hybrid nanofibrous scaffolds developed by Shrestha et al. [119] are a promising platform for bone tissue engineering. The use of integrated zein and chitosan (CS) with polyurethane (PU) and functionalized multiwalled carbon nanotubes (fMWCNTs) results in the creation of a material that mimics the natural extracellular matrix. Studies have shown that these scaffolds, especially those containing 0.1 mg/mL fMWCNTs, are characterized by significant improvement in mechanical properties, hydrophilicity, and antibacterial efficiency. These features are crucial for creating an environment conducive to bone cell growth. Additionally, the presence of fMWCNTs stimulates biological molecules, which contributes to the activation of osteogenesis processes and prevents bone resorption. The results of the studies indicate that the hybrid scaffolds promote rapid intercellular communication through a biointerface and support the regeneration of pre-osteoblasts in vitro, leading to their growth, proliferation, and differentiation. In addition, these scaffolds showed the ability to nucleate hydroxyapatite (HY) nanocrystals and express osteogenic differentiation markers such as osteopontin and osteocalcin. This suggests their potential as bone tissue engineering materials. However, it should be noted that despite promising in vitro results, there is a lack of in vivo studies that could confirm the efficacy and safety of these scaffolds (containing nanoparticles—CNTs) in clinical applications.

In recent years, the development of 3D printing technology has significantly influenced bone tissue engineering, enabling the creation of innovative bone substitutes. Thanks to the flexibility of this method, it is possible to produce structures with complex geometry, which is crucial in the context of adapting implants to individual patient needs. Three-dimensional printing allows for precise control over the micro- and macrostructure of materials, which is particularly important in the case of porosity. Appropriate porosity is essential for the integration of the implant with natural bone tissue and for ensuring adequate permeability to fluids and cells, which favors the regeneration process.

**Figure 9:**
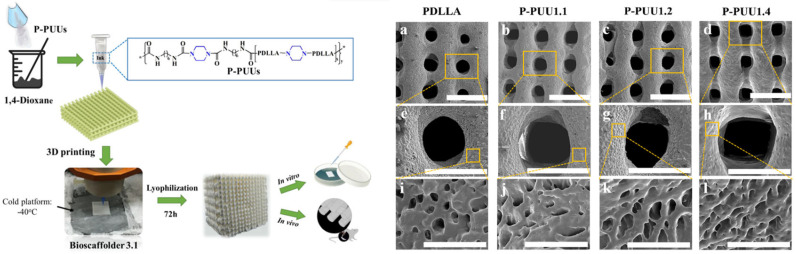
Schematic illustration for preparation of polymeric scaffolds by air-driven extrusion 3D printing assisted with a low-temperature receiver (left side) and surface morphology and macro-/micropores of polymeric scaffolds (macropores size of about 450 μm; micropores size of 2–10 μm) (bar—(**a**–**d**): 1 mm; (**e**–**h**): 500 μm; (**i**–**l**): 50 μm) (right side). Reproduced with permission from ref. [122], Copyright 2019, ACS Publications.

Additionally, the elimination of contamination problems that can occur in traditional manufacturing methods makes 3D printing more attractive. The ability to directly print from various materials, such as metals and ceramics, opens up new possibilities in the design of implants that can be more functional and adapted to specific clinical conditions [120]. Three-dimensional printing ink materials should show degradability, biocompatibility, and processability, and their degradation products must not have side effects on human health [121]. For the first time, Ma et al. [122] developed a series of biodegradable piperazine (PPZ)-based polyurethane–urea (P-PUU) scaffolds with a gradient of PPZ content by pneumatic extrusion 3D printing technology. They demonstrated that the P-PUU ink at 60 wt% had a suitable viscosity for fabricating the scaffolds. The 3D-printed P-PUU scaffolds showed an interconnected porous structure with a macropore size of about 450 μm and a porosity of about 75%—Figure 9.

By adjusting the PPZ content of the P-PUU scaffolds, their mechanical properties could be moderated, and the scaffolds with the highest PPZ content showed the highest compressive modulus (155.9 ± 5.7 MPa) and strength (14.8 ± 1.1 MPa). Furthermore, both in vitro and in vivo biological results suggested that the 3D-printed P-PUU scaffolds possessed excellent biocompatibility and osteoconductivity, which facilitated new bone formation.

**Figure 10:**
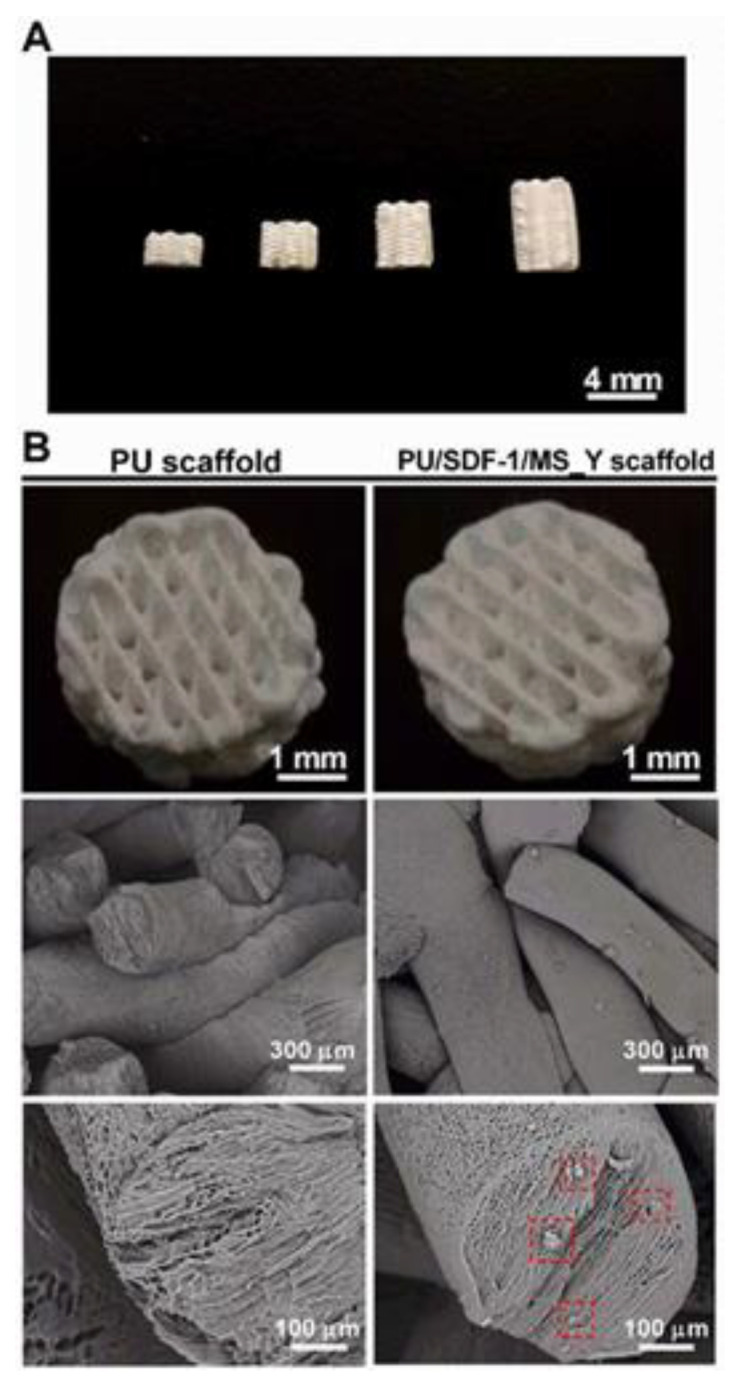
(**A**) The side view of the PU scaffolds printed with 200 ng/mL SDF-1 and 55 µg/mL Y27632-loaded microspheres (PU/SDF-1/MS_Y), with various heights. (**B**) The top views of the 3D-printed scaffolds and the SEM images for the microstructure of the scaffolds and for each stacking fiber. The box in (**B**) indicates the presence of microspheres (MS_Y). Reproduced with permission from ref. [123], Copyright 2019, Elsevier.

In the research conducted by Wen et al. [123], an innovative 3D printing ink was developed that combines a biodegradable water-based polyurethane (PU) dispersion with the chemokine SDF-1 and microspheres embedded in Y27632. The aim of this solution was to create scaffolds that offer the function of sequential release of biological substances. The PU microspheres that were incorporated into the ink act as a carrier, enabling the controlled release of Y27632. During the printing process, the scaffolds are formed at low temperature (Figure 10), which allows the first release of SDF-1. This chemokine stimulates cell migration into the scaffolds. Then, after a certain period of time, the Y27632 microspheres are released, which induces cell differentiation towards chondrocytes without the need for prior cell seeding. Optimized PU scaffolds were tested in the context of cartilage regeneration, and the results obtained show that they can be directly implanted into cartilage–bone defects in rabbits, indicating thus their potential in cartilage tissue regeneration.

Adhami et al. [124] analyzed 3D-printed scaffolds made of thermoplastic polyurethane (TPU) and enriched with dipyridamole (DIP). The aim of their work was to evaluate the osteogenic capacity of these scaffolds in vitro. The studies focused on the DIP release profiles, biocompatibility of the scaffolds with pre-osteoblastic cells derived from mouse skull, and intracellular alkaline phosphatase (ALP) tests to verify the osteogenic capacity and assess mechanical properties. It has been found that the addition of DIP at concentrations up to 10% *w*/*w* did not affect the compressive modulus of the scaffolds, and the tested materials were able to continuously release the drug for up to 30 days. Moreover, a significant increase in cell viability, proliferation, and osteogenesis of MC3T3-E1 cells was observed at the highest DIP loading (10% *w*/*w*) compared to the control samples. These promising results suggest that TPU-based scaffolds loaded with DIP can support bone regeneration. Due to the flexibility of 3D printing technology, this approach has the potential to create customized scaffolds that can be tailored to individual patient needs, which could have significant implications for future medical practice.

The 3D-printed SMPU/Mg composite scaffolds developed by Zhang et al. [125] represent a novel approach to address the challenges associated with traditional shape memory implants, such as low porosity and unsatisfactory mechanical and bioactive properties. The use of shape memory polyurethane (SMPU) and magnesium (Mg) as a photothermal and bioactive component allowed the creation of a scaffold that combines several beneficial features. The SMPU/4 wt% Mg scaffold exhibits a hierarchical porous structure, which promotes better nutrient absorption and supports regenerative processes. Compared to the traditional SMPU scaffold, the new composite scaffold is characterized by a higher compressive strength (~6.7 MPa vs. ~5.9 MPa) and higher modulus (~23.0 MPa vs. ~16.8 MPa), making it more suitable for tissue engineering applications. The key innovation is the ability of the scaffold to respond to NIR light, which allows for a rapid increase in temperature and activation of the shape memory effect. Thanks to Mg particles, which have photothermal properties, the scaffold can effectively regain its shape. Moreover, in vitro studies have shown that the scaffold supports cell survival, proliferation, and osteogenic differentiation, which is crucial for bone regeneration processes. Additionally, animal studies have confirmed the osteoprotective functions of the scaffold and its ability to create tight contacts with the surrounding tissue. The use of low-temperature 3D printing technology (LT-RP) has enabled the creation of a complex porous structure, which is difficult to achieve with traditional manufacturing methods. Finite element analysis indicates a significant relationship between the tight contact of the scaffold and the shape recovery process, which emphasizes its innovativeness.

## 6. Soft-Tissue Engineering

### 6.1. Skeletal Muscle Tissue Engineering

Skeletal muscles are the major muscles in our body and have various critical functions [126].

Skeletal muscles make up to 40% of the total body mass. Not only do they play a key role in our movements, but they also keep our bodies in a state of balance by regulating energy production. One of the most important physiological features that help maintain this balance is the extraordinary ability of skeletal muscles to adapt to changed functional needs. On a macro scale, such adaptation is clearly seen in athletes, marathon runners, and weightlifters. While marathon runners’ training focuses on strengthening fatigue-resistant muscles, weightlifters’ training focuses on working on muscles that react quickly and produce a lot of power. A number of disorders can severely impair normal muscle function. In particular, traumatic skeletal muscle injury can be caused by bruising, extreme cold or toxins, road accidents, surgery, or sports-related injuries [127]. Up to 55% of all sports injuries result in damage at the muscle fiber level [128]. Loss of more than 20% of total muscle mass results in reduced function and impaired tissue regeneration [129]. Despite the impressive ability of the body to regenerate on a daily basis, in some cases, skeletal muscle cannot be fully rebuilt. In the case of minor damage, the muscle can recover completely, but when the damage is significant, repair of large losses of muscle mass may be impossible, and the damage is irreversible. This makes rebuilding and improving muscle function one of the greatest biomedical challenges of our time. Tissue-engineered muscle represents a suitable strategy for replacing damaged or lost muscle tissue. A particular challenge, however, is producing structures that accurately mimic the mechanical properties of muscle, such as soft and flexible woven fabrics that can withstand large uniaxial loads and stretch up to 60% before mechanical failure [130].

#### Polyurethane Scaffolds for Skeletal Muscle Engineering

In order to design scaffolds that mimic the mechanical properties of skeletal muscle, different PU formulations were investigated as skeletal muscle scaffolds, obtained by different techniques, i.e., freeze-drying, bioprinting, decellularization, and electrospinning [126].

Chen et al. [131,132] synthesized biodegradable, electroactive, and elastic polyurethane–urea copolymers from amine-capped aniline trimer, dimethylol propionic acid, polylactide, and hexamethylene diisocyanate. The high breaking elongation rate of these poly(urethane urea) (PUU) copolymers (between 145.2% and 641.7%) is attributed to the hydrogen bonds between the ester, urethane, and urea groups which serve as physical cross-linking points to enhance the intra- and intermolecular interaction between the PUU macromolecules. These materials have also been shown to have an enhanced effect on myogenic differentiation of C2C12 myoblasts.

Along this line of interest, Xu et al. [133] reported the incorporation of aniline trimer into polycaprolactonediol-based polyurethane (CPU) and then doped with camphorsulfonic acid (CSA). The electrical conductivity was studied under both dry and wet conditions. The results showed that wet CPU films exhibited better conductivity compared to dry film samples. Increasing the CSA content in the films also increased the electrical conductivity of the obtained materials.

Random block polyurethanes (PULA-ran-3/4HBs) and alternating block polyurethanes (PULA-alt-3/4 HBs) based on the polyester poly(lactic acid) (PLA) and poly(3-hydroxybutyrate-co-4-hydroxybutyrate) (P3/4HB) copolymer were studied by Niu [134]. The porous PULA-alt-3/4 HB scaffolds showed hemocompatibility, in vitro and in vivo cytocompatibility, and hydrophilicity due to a more regular structure compared to PULA-ran-3/4 HB materials, making them a better candidate for muscle tissue scaffolds. Implantation in rats for six weeks was successful, and no inflammation associated with muscle tissue formation was observed in the case of alternating and random copolymeric scaffold structures.

In recent years, significant progress has been observed in electrospinning techniques, which make it possible to obtain scaffolds in the form of biomimetic nanofibrous morphologies consisting of fibers similar to fibrous ECM proteins. Such topography affects cell proliferation, adhesion, and phenotype [135,136,137].

The electrospinning technique was used by Yıldırım et al. [126], who obtained nanofibers based on thermoplastic polyurethane (TPU) doped with clinoptilolite (CLN). Mechanical tests showed that pure TPU and TPU doped with 5 and 10 wt% CLN showed Young’s modulus values of 3.66, 2.37, and 1.85 MPa, respectively, while the elongation at break was 118.37%, 106.18%, and 179.0%, respectively. Cytotoxicity tests showed that the obtained TPU/CLN membranes are biocompatible, and cell adhesion increases proportionally with the increase in the amount of CLN in the composite.

Artificial muscles based on electrospun polyurethane (PU) microfiber yarns embedded with carbon nanotubes (CNTs) have been studied in detail by Meng et al. [138]. Due to the presence of nanotubes in the yarns, these materials show the ability to effectively absorb near-infrared radiation and heat. This phenomenon leads to rapid temperature changes, which in turn results in contractions and expansions along the axial direction of the yarn. These yarns made of microfiber mats are highly twisted into a coiled form, which allows for a maximum shrinkage of 6.7% at 70 °C. Importantly, the tests carried out showed that the material is able to withstand 1000 cycles of contractions without noticeable loss of performance, suggesting their high durability. The solution blow spinning (SBS) technique was used to produce fibrous polyurethanes with fiber diameters of 200, 500, and 1000 nm [139]. The effect of the collector rotation speed on the fiber alignment and diameter was investigated, and it was shown that the larger the fiber diameter, the better controlled architectures can be obtained, and that the scaffold pore size increased with increasing fiber diameter, but decreased with increasing fiber alignment. Mechanical property studies showed that they strongly depend on the stretching direction, but fiber orientation affects mechanical strength only in the case of materials with a fiber diameter of 1000 nm. On scaffolds with aligned fibers and the largest fiber diameter (1000 nm), pericyte growth occurred. Scaffolds obtained by the SBS technique can be used for controlled design and production of scaffolds with aligned fibers [140].

Traditional methods such as electrospinning or freeze-drying have limitations, especially when it comes to producing realistic, large-scale muscle grafts. These techniques often require secondary colonization to obtain larger constructs, which complicates the even distribution of cells and makes it difficult to ensure a homogeneous population of different cell types. Among the various available fabrication techniques, 3D bioprinting stands out for its precision in creating the complex architecture required for efficient skeletal muscle. This method allows for the deposition of cell-laden hydrogels in a controlled, layer-by-layer manner, effectively mimicking the extracellular matrix (ECM) environment [141,142,143].

**Figure 11:**
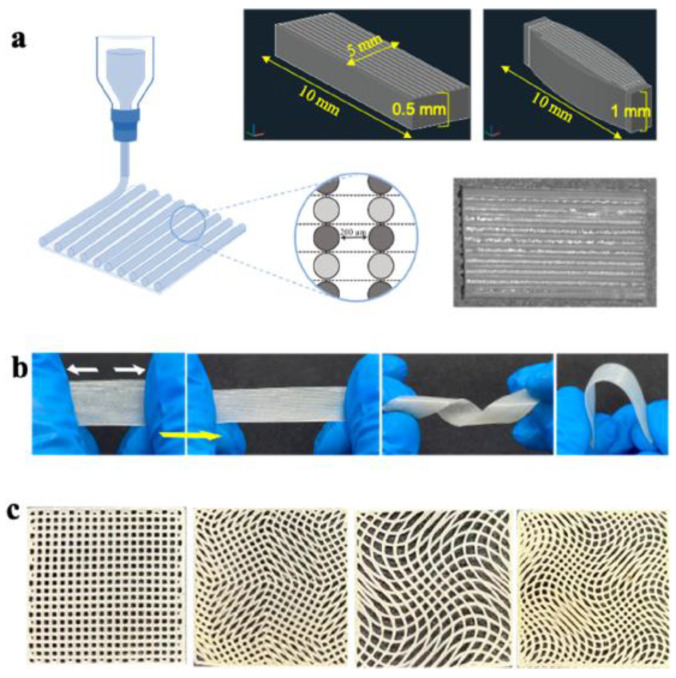
(**a**) Set-up for 3D printing of TPU scaffolds. Scaffolds were prepared in rectangular and spindle-shaped outer geometry for in vitro and in vivo applications, respectively. Inner structures of both scaffold types were made of parallel-aligned fibers in 3D. (**b**) Representative images of 3D-printed TPU scaffolds showing elasticity and recovery of the materials upon deformation. (**c**) 3D-printed TPU scaffolds with different architectures that closely comply with the 3D model. Reprinted from ref. [144], Copyright 2021, ACS Publications.

The study by Gokyer et al. [144] focused on the development and evaluation of a new 3D-printed scaffold made of thermoplastic polyurethane–urea (TPU) elastomer that was designed to mimic the mechanical properties of skeletal muscle extracellular matrix (ECM). This was particularly important due to the limitations of other synthetic biomaterials, such as polycaprolactone (PCL), which showed significantly reduced elasticity (28% elongation) compared to the TPU scaffold (940% elongation). The TPU scaffold was designed with a parallel internal architecture to improve cellular behavior—Figure 11. In vitro experiments demonstrated successful seeding and differentiation of C2C12 myoblasts and human adipose-derived stem cells (hADSCs) on the scaffold, a crucial step before moving on to in vivo applications.

In subsequent experiments using a rat model of volume muscle loss (VML), Gokyer et al. [144] compared the performance of cell-free scaffolds with that of cell-loaded scaffolds containing hADSCs. The results showed that the cell-loaded scaffolds promoted significant muscle tissue regeneration, while the acellular scaffolds did not show similar results. Furthermore, the study showed that the regenerated muscle tissue was able to generate force exceeding preoperative levels by 112%, which highlights the high potential of the TPU scaffold in muscle tissue engineering applications. Overall, the results of this study highlight the advantages of using TPU over traditional biomaterials for muscle regeneration, especially in terms of appropriate mechanical properties and enhanced bioactivity in combination with appropriate cell types. This work represents a significant step forward in the field of regenerative medicine and muscle tissue engineering, offering promising opportunities for future research and clinical applications.

### 6.2. Cardiovascular Tissue Engineering

Cardiovascular tissue engineering (CTE) is a rapidly developing field that focuses on the creation and regeneration of tissues of the heart and vascular system to improve the treatment of cardiovascular diseases and increase the quality of life of patients. CTE uses various approaches, including stem cells, which have the ability to differentiate into various cell types and to form novel biomaterials, which serve as scaffolds to support the growth of new tissues. The main goals of CTE are:

- Blood vessels: Creating bio-engineered blood vessels that can replace damaged or blocked vessels is crucial to restoring proper blood circulation. The use of biodegradable scaffolds and hydrogels allows for the development of a structure that not only provides physical support, but also promotes integration with existing tissue.
- Heart muscle: Regeneration or replacement of damaged heart muscle after heart attacks or other diseases is an important goal. Research into stem cells and various biomaterials for tissue engineering could lead to new therapies that speed healing and improve heart function.
- Heart valves: When heart valves are damaged or failing, tissue engineering could offer the potential to create new, biological valves that are better tolerated by the body and have a lower risk of rejection [145,146,147].

The most commonly used biomaterials in CTE are biodegradable scaffolds (offer mechanical support in the initial phases of regeneration and gradually degrade in the body, allowing for the formation of natural tissue), hydrogels (show properties resembling soft tissue and are used to support cell growth and differentiation, which is important in the context of heart muscle formation), and decellularized tissue (based on the removal of cells from natural tissues, preserving their extracellular matrix, which can stimulate regeneration and integration with new cells) [148,149].

Cardiovascular tissue engineering has the potential to transform the treatment of heart disease, offering alternatives to traditional surgical procedures and transplantation. With advances in science and technology, CTE has the potential to become a significant tool in providing better healthcare and quality of life for patients suffering from cardiovascular disease. Developing new tissue engineering and regenerative medicine methods for reconstructing the heart ventricular wall is a promising research direction. Approaches such as the use of flexible, biodegradable patches that mimic the natural structure of the heart muscle could have a significant impact on the treatment of patients after heart attacks. While the heart muscle is characterized by a unique fiber arrangement, and its functionality relies on the ability to contract spontaneously, damage caused by infarction leads to many adverse changes. Left ventricular remodeling after infarction is a serious problem that includes not only mechanical dilation of the ventricle, but also changes in shape and hypertrophy of the heart muscle. These processes are often associated with declining heart function and can lead to heart failure, which deprives patients of their quality of life and increases the risk of death [149].

The use of modern bioengineered materials, such as specially designed patches, can help improve heart mechanics after heart attacks. Such solutions can support tissue regeneration, improve contractile function, and have a positive impact on the overall health of patients. Continued research in this area is crucial to introducing innovative therapies that can impact clinical outcomes and the survival of patients with heart disease [150].

#### Polyurethane Scaffolds for Cardiac Muscle Engineering

Cardiac patches, as an innovative solution in heart regeneration, play a key role in the therapy of myocardial injuries. Made of various materials, both natural and synthetic, they aim to mimic the natural structure of heart tissue. Thanks to the microstructure that mimics the biological environment, these patches can support cell adhesion and proliferation, which promotes tissue regeneration. Adapting the patch to specific needs, such as size, shape, and mechanical strength, is extremely important to ensure its compatibility with the surrounding tissues. Also, the possibility of seeding cells in the patches before their implantation opens up new possibilities in the context of heart regeneration, allowing their development and differentiation in a controlled culture environment. It is crucial that these materials can degrade in sync with the natural healing process, helping to avoid problems such as the formation of a fibrous capsule that could lead to a chronic inflammatory response [151].

Adapting the mechanical properties of materials to the characteristics of the heart is important because the stiffness of cardiac cells changes during the cardiac cycle. Stiffness values that start from lower values in early diastole (10–20 kPa) and reach much higher values in late diastole (200–500 kPa) indicate that materials used in regeneration should reflect these changes to support proper cell contraction and their integration with the surrounding tissue [152]. With such approaches, cardiac patches have the potential to improve myocardial function and support repair processes after heart attacks or other cardiac injuries [153].

**Figure 12:**
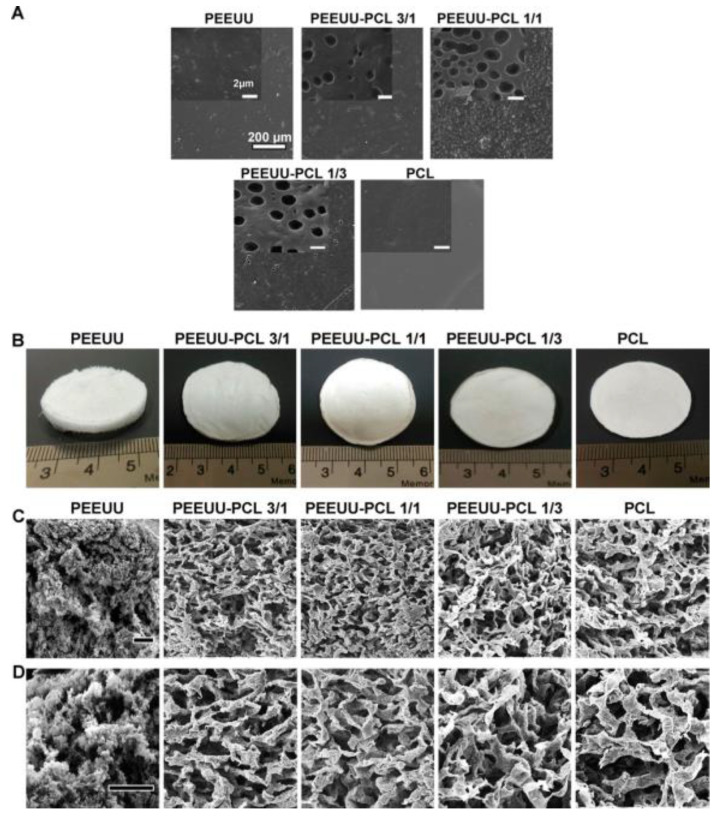
(**A**) SEM micrographs of the fabricated films. (**B**) Macroscopic images and (**C**,**D**) SEM images of the scaffolds fabricated by the solvent casting and salt leaching methods. (**C**,**D**) Micrographs with low and high magnification, respectively. Scale bars: 200 μm. Reprinted from ref. [154], Copyright 2018, ACS Publications.

The research conducted by Asadpour et al. [154] concerned the synthesizing of biodegradable poly(ester–ether–urethane–urea) (PEEUU), which was blended with polycaprolactone (PCL)—Figure 12—to improve its mechanical and physicochemical properties.

As a result of this process, it was found that the elasticity of the fabricated blends increased with the increasing share of PEEUU. The studies observed that the elastic moduli reached various values: for PEEUU, it was 0.62 ± 16 MPa; while for PEEUU-PCL in the proportion of 3/1, it was 12.62 ± 3.1 MPa; and in the proportion of 1/1, it reached 63.3 ± 4.07 MPa. However, the modulus of elasticity of PEEUU films and PEEUU-PCL blends was in the range of values similar to that of native heart tissue, making them promising materials in the context of medical applications. The low stiffness of the resulting materials may be beneficial for integration with native heart tissue, because a too-stiff polymeric blend may inhibit cardiomyocyte growth and function. Additionally, blending PEEUU with PCL showed good biocompatibility. These blends revealed appropriate cell–scaffold interactions, resulting in the increased functional activity of cardiomyoblasts, which could be measured by analyzing the expression of markers specific for heart tissue. These results suggest the potential of using PEEUU-PCL in the context of cardiac tissue regeneration.

Baheiraei et al. [155,156] obtained electroactive polyurethane/siloxane films containing fragments of aniline tetramer. The presence of electroactive aniline tetramer in these materials leads to increased expression of heart-specific genes, which is crucial for cardiac contractile function and electrical signaling. Increased expression of genes such as Cx43 (connectin cadherin), TrpT-2 (receptor protein), and SERCA (calcium pump protein) suggests that these changes may support better intercellular connections and synchronized contractions, which are crucial for efficient cardiac muscle function. Furthermore, these materials were shown to be biocompatible and do not negatively affect the intrinsic electrical properties of HL-1 cells. Incorporation of the electroactive part of polymeric constructs improves their ability to transmit electrical signals between cells.

Electroconductive nanofibrous polyurethane/graphene (PU/G) composites obtained using different concentrations of graphene and two different fabrication techniques (electrospinning and solvent casting) showed interesting electrical and mechanical properties. Research by Bahrami et al. [157] revealed that with increasing graphene content in the materials, their electrical conductivity increased significantly. Graphene acted as electrical bridges, which contributed to the improvement of the electrical conductivity of PU/G composites. Additionally, the mechanical properties of composites obtained by electrospinning were significantly better compared to those produced by solvent casting. Biological evaluations confirmed that the introduction of graphene supported cell adhesion and proliferation, suggesting that these composites are non-toxic and can be used in biological and medical applications. The study results indicated that graphene was better distributed in the polyurethane matrix when the electrospinning method was used, which may have a significant impact on the final mechanical and electrical properties of the composites.

In the work of Mani et al. [158], an electrospun new nanocomposite filled with basil oil and titanium dioxide (TiO_2_) particles was developed. The composite material showed increased hydrophobicity and reduced fiber diameter compared to the original polymer. The prepared patch delayed the clotting time and provided a safe environment for red blood cells, which indicates its hemocompatibility, and the cellular toxicity of the developed composite was lower than that of the original polymer.

The research conducted by Tao et al. [159] focused on the use of an innovative angiogenic hydrogel based on poly(ethylene glycol) fibrin, reinforced with a biodegradable poly(ether–urethane)urea (BPUR) mesh, for the development of ventricular heart wall replacement in rats. The developed hydrogel scaffold aimed to stimulate cell invasion and the formation of new blood vessels, which is crucial for tissue regeneration. During the research, a significant improvement in regenerative tissue remodeling was observed in places where an artificial defect on the right ventricular wall was introduced. The use of an engineered hydrogel patch reinforced with BPUR contributed to the intensification of blood vessel formation and muscle regeneration, which indicates its potential effectiveness in the treatment of heart damage. Due to the limited fibrosis reaction, a reduced number of arrhythmia cases and improved heart performance were observed in the studied animals. These results suggest that the developed hydrogel scaffold may have significant implications for future therapies aimed at regenerating and improving heart function following heart injury.

The research work of Chen at al [160] presents a biomimetic electrospun anisotropic scaffold based on polyurethane (PU) and ethylcellulose (EC), characterized by uniform fibrous nanostructures and three-dimensional porous networks. The results showed that the anisotropic folds of the scaffolds with aligned nanofibers were successfully fabricated and exhibited high mechanical properties for cardiac cell survival and function.

Immediately after myocardial infarction, there is an overproduction of reactive oxygen species (ROS), which leads to disruption of cellular homeostasis. These disorders contribute to cardiomyocyte damage and promote inflammatory processes and fibrosis in the heart. ROS play a key role in the pathological processes of cardiac remodeling after infarction, associated, for example, with increased activity of nicotinamide adenine dinucleotide phosphate (NADPH) oxidase, which is one of the main sources of these reactive species [161].

**Figure 13:**
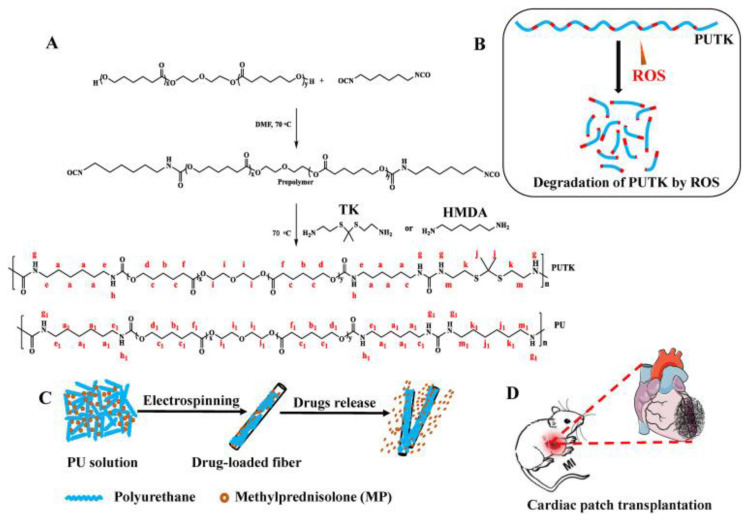
(**A**) Synthetic scheme for ROS-responsive polyurethane (PUTK) and non-responsive polyurethane (PU) from poly(ε-caprolactone) diol, 1,6-hexamethylene diisocyanate, and chain extenders of ROS-cleavable thioketal (TK) and 1,6-hexanediamine (HMDA), respectively. (**B**) The thioketal linkers in PUTK are cleaved in response to reactive oxygen species (ROS) expressed by injured or inflamed tissues in situ. (**C**) Fabrication of electrospun fibrous patches loaded with methylprednisolone (MP) for sustainable release. (**D**) Transplantation of the cardiac patch on the surface of myocardial infarction (MI) for restoring the structures and functions of infracted myocardium. Reprinted from ref. [161], Copyright 2020, Elsevier.

The study by Yao et al. [161] focused on biodegradable elastomeric ROS-responsive polyurethane containing thioketal bonds (PUTK). This material was synthesized from polycaprolactonediol (PCL-diol), 1,6-hexamethylenediisocyanate (HDI) and a ROS-cleavable chain extender. PUTK was electrospun into fibrous patches that were capable of loading the glucocorticoid methylprednisolone (MP)—Figure 13. These patches were used to treat myocardial infarction (MI) in rats in vivo. In long-term treatment lasting 28 days, the fibrous patches containing MP significantly improved cardiac function and angiogenesis, and effectively reduced fibrosis and negative cardiac remodeling after MI. The results of these studies suggest that PUTK may have great potential to optimize and promote its use in the treatment of myocardial infarction.

One of the major limitations of electrospinning is the difficulty in obtaining large pore sizes in the fabricated structures. Typically used electrospinning techniques focus on creating fibers with very small diameters, which can lead to microscale pore sizes [162].

To overcome these problems, scaffolds have been prepared by the thermally induced phase separation (TIPS) technique, which allows the control of the scaffold pore size by changing the preparation conditions [163].

**Figure 14:**
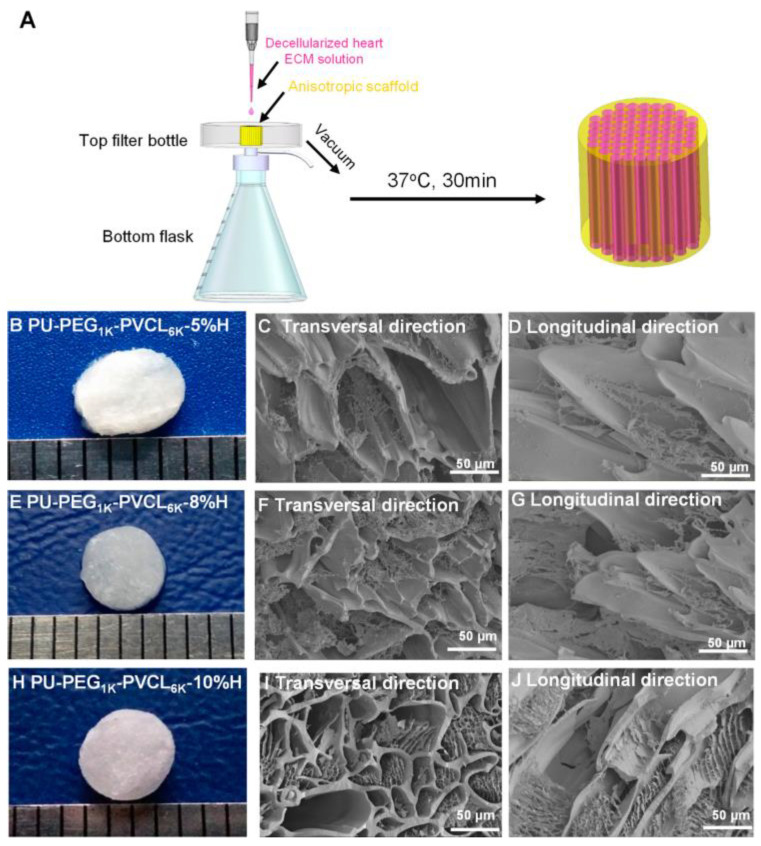
Biohybrid scaffolds fabricated by combining PU-PEG1K-PVCL6K anisotropic scaffolds (5%, 8%, and 10% *w*/*v*) with myocardium-derived hydrogel. (**A**) Decellularized heart ECM solution was added dropwise onto the synthetic anisotropic scaffold and then absorbed into the scaffold by low vacuum. After placing the ECM–scaffold complex at 37 °C for 30 min, the biohybrid scaffold was formed. (**B**,**E**,**H**) Digital images of PU-PEG1K-PVCL6K-5% H, PU-PEG1K-PVCL6K-8% H, and PU-PEG1K-PVCL6K-10% H. (**C**,**F**,**I**) Electron micrographs of PU-PEG1K-PVCL6K-5% H, PU-PEG1K-PVCL6K-8% H, and PU-PEG1K-PVCL6K-10% H biohybrid scaffolds in the transversal direction. (**D**,**G**,**J**) Electron micrographs of PU-PEG1K-PVCL6K-5% H, PU-PEG1K-PVCL6K-8% H, and PU-PEG1K-PVCL6K-10% H biohybrid scaffolds in the longitudinal direction. Reprinted from ref. [164], Copyright 2020, Elsevier.

Xu et al. [164] described an advanced approach to designing biomedical scaffolds made of polyurethane (PU-PEG-PVCL) that were optimized for mechanical adaptation to the native myocardium. The authors used the TIPS method to synthesize an anisotropic scaffold, which allowed for tuning the mechanical properties of the material by manipulating the polymer concentration and selecting appropriate soft segments, which were triblock copolymer of poly(ethylene glycol) (PEG), random copolymers of ε-caprolactone (CLs), and δ-valerolactone (VL). The aim was to achieve mechanical parameters of the designed scaffold that would resemble those of native myocardium. The authors focused on aspects such as suture retention strength, uniaxial and biaxial mechanical properties, and fracture toughness. The results showed that the optimal scaffold achieved a fracture strength of 20.7 ± 1.5 N, which is comparable to that of native cardiac tissues (20.4 ± 6.0 N). Additionally, the biaxial mechanical stretching of the scaffold was similar to that of the myocardium. Analyzing the possibility of creating innovative biomaterials, the authors combined the optimized scaffold with a hydrogel derived from porcine myocardium, which resulted in the creation of a biohybrid scaffold with a morphology resembling a decellularized myocardium matrix (Figure 14). The SEM images of the obtained biohybrid scaffolds showed the fibrous myocardium ECM distributed on the surface and inside their aligned pores.

In experiments after implantation of such a scaffold in rat models, minimal immune response and improved cell penetration were demonstrated compared to PU scaffolds used alone. The studies conducted underline the advantages of the biohybridization of synthetic polymers from biological tissues, which allows for the preservation of favorable tissue properties, such as native extracellular matrix (ECM) components and their biological activity, while obtaining durable and mechanically strong synthetic materials.

### 6.3. Cardiac Tissue Engineering

The heart is a key organ in the circulatory system, responsible for pumping blood to various parts of the body. Heart valves play an important role in controlling blood flow and preventing its backflow. In the event of damage or disease, valve replacement becomes necessary. Artificial heart valves are an essential element of modern cardiology. Mechanical valves, made of materials such as carbon or metals, are very durable but require long-term use of anticoagulants, which is associated with the risk of bleeding. Biological valves, on the other hand, derived from animal tissue are more biocompatible, which means a lower risk of complications related to clotting, but their durability is limited. They often calcify or degenerate over time, which may lead to the need to replace them [53].

Since the first polymeric heart valves (PHVs) implanted in the 1960s, which were made of polyurethane and then, due to material and technological limitations, did not meet the expectations in terms of durability, scientists have been constantly looking for more innovative solutions. Over the past decades, many different materials and structures have been tested, including the use of molecular design techniques and modifications of existing polymers. In addition, research on additional components such as nanomaterials has become a promising direction that could improve the mechanical properties and biocompatibility of valves. However, despite the progress, polyurethane valves have still not achieved sufficient durability and resistance to degradation, which limits their clinical use. Problems such as the deposition of calcium salts, leading to calcification and reduced valve function, remain a key challenge to overcome. There is still much room for development and improvement of polymer heart valves, and further research is needed to transform these theoretical concepts into practical, effective solutions that can improve the outcomes of patients with heart diseases [165].

#### Polyurethane Scaffolds for Cardiac Valve

The literature review showed that three polyurethane-based heart valves have been intensively studied over the last decade and have either been clinically tested or are currently being tested. These are the TRISKELE transcatheter valve, SiPUU (LifePolymer), and the first implantation of these PHVs in humans [166], and FGO-PCU (Hastalex^®^) [167], a unique polymer with incorporated graphene [168]. The TRISKELE transcatheter valve made of POSS-PCU is a trileaflet valve. It consists of a nitinol wire frame, leaflets, a POSS-PCU sealing cuff, and a skirt supporting the sealing cuff. The structural elements of the TRISKELE valve were manufactured separately.

POSS-PCU is a nanocomposite polymer consisting of a hard crystalline segment and soft elastomeric segments, in which polyhedral oligomeric silsesquioxane (POSS) nanoparticles are attached as pendant chain functional groups to a poly(carbonate–urea)urethane (PCU) backbone. In vitro studies of this polyurethane have shown increased hemocompatibility and a surface resistant to thrombogenesis [169], biostability [169], and resistance to calcification [170]. The described study focuses on an innovative approach to the production of heart valves, using materials such as POSS-PCU in combination with stainless steel. The production method consisting in immersing the stem in a polyurethane solution allows for obtaining uniform and repeatable hydrodynamic properties (PHVs), with a controlled leaflet thickness of 130 ± 10 µm on average. The valve prototypes were tested in different sizes (23, 26, 29 mm) under simulated anatomical conditions, which allowed for checking their functionality in the context of the aorta. The results showed that the TRISKELE valves are characterized by hydrodynamic performance that is at least comparable to the currently used reference models. An additional advantage of the TRISKELE valves is a significant reduction in paravalvular leakage, which is crucial for the safety and effectiveness of implantation. Better anchoring and sealing also indicate the high potential of the TRISKELE valves. The valve is currently in preclinical development [171].

SiPUU is a polyurethane in which the soft segments are based on α,ω-bis(6-hydroxyethoxypropyl)poly(dimethylsiloxane) and poly(hexamethylene oxide) (PHMO), while the hard segments are 4,4′-methylenediphenyldiisocyanate (MDI) and a mixture of 1,3-bis(4-hydroxybutyl)-1,1,3,3-tetramethyldisiloxane (BHTD) and 1,2-ethylenediamine (EDA). In vitro studies have shown that the newly developed polymer is characterized by high oxidation resistance, which is important in the context of its use in a physiological environment. The presence of dimethyl siloxane (PDMS) affects the structure of the polymer, increasing its durability and resistance to environmental factors. Ex vivo thrombogenicity tests have shown that the SiPUU polymer causes virtually no platelet adhesion or clot formation on its surface. Additionally, long-term implantation of the SiPUU valve prototype in sheep did not reveal any differences in thrombogenicity or tissue response compared to the conventionally used biological valve. SiPUU (LifePolymer) valves consist of three main elements: a stent, two or three specially designed leaflets, and a sewing cuff. The leaflets are formed in solution on stents made of polyetheretherketone and then mechanically processed for precise dimensional adjustment. This approach allows for obtaining efficient and functional heart valves that can be widely used in medicine [168].

Foldax^®^, under the trade name Tria™, recently conducted a first-in-human clinical trial of 15 patients undergoing surgical replacement of the LifePolymer valve [169,170,171,172]. The results of this study suggest that most of the complications observed were not related to the valve chosen or to the surgical procedure itself. However, some results suggested that the occurrence of clots may have been related to the Tria valve suture ring, which is made of polytetrafluoroethylene rather than polyurethane. The polysiloxane-based valve demonstrated good hemodynamic function in this first-in-human experience, which is a positive sign. However, due to the limited size of the population and the need for long-term monitoring, further studies with a larger group of patients are needed to assess the efficacy and safety of this type of valve over a longer period of time.

**Figure 15:**
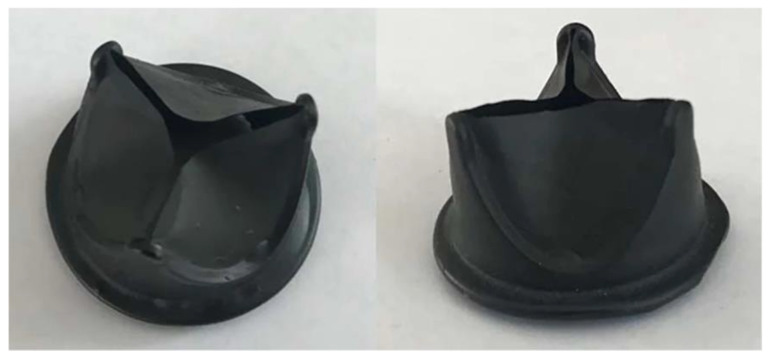
A prototype of the polymeric heart valve made from Hastalex. Reprinted from ref. [167], Copyright 2020, Springer Nature.

In [167], a detailed evaluation of a nanocomposite consisting of functionalized graphene oxide and poly(carbonate–urea)urethane, known under the brand name “Hastalex”^®^, was made—Figure 15.

It was compared with GORE-TEX™, a commercial polymer traditionally used in medicine, especially in the context of treating cardiovascular diseases. One of the key factors influencing the high tensile strength of Hastalex is the presence of graphene oxide in the polymer matrix. The high elasticity of this polymer allows the production of thinner leaflets, making it a promising material for applications in heart valves, in particular in transcatheter valve replacements (TVRs). Cytotoxicity tests indicate the high biocompatibility of FGO-PCU, which is further confirmed by the results of tests on cell adhesion, viability, and proliferation. Despite of the promising results, there is currently only one prototype of a heart valve prosthesis made of Hastalex polymer, and there are no data on its hydrodynamic performance and in vivo test results. This indicates the need for further research and analysis before the wide application of this new material in clinical practice [173].

Recently, for the first time Melo et al. [174] developed new environmentally friendly isocyanate-free polyurethanes (NIPUs) as potential biomaterials for the production of synthetic heart valve prostheses. NIPUs are a “green” alternative to traditional polyurethanes, and their low thrombogenic profile makes them promising for use in medicine. When used for the production of valve elements, tested in laboratory conditions, they showed hydraulic properties comparable to those used in clinically recognized bioprostheses, which suggests their high efficiency and safety. Additionally, the resistance to calcification of patches with NIPU is another advantage that may contribute to the longer service life of these implants. This study provides a basis for the future development of isocyanate-free, hemocompatible and hydrodynamically competent heart valve prostheses made from NIPU.

### 6.4. Vascular Tissue Engineering

Vascular tissue engineering is an important field of research that aims to develop innovative solutions to overcome the disadvantages of conventional blood vessel substitutes [175].

Blood vessels are part of the circulatory system, and their function is to transport blood throughout the body. Blood circulation in the human body is provided by three types of blood vessels: arteries, veins, and capillaries. They perform different functions, which translates into differences in their structure. Arteries, which are responsible for carrying blood away from the heart at high pressure (90–70 mmHg; 8–12 MPa), have thicker walls than veins, which direct blood at lower pressure (15–20 mmHg; 2–2.7 MPa) back to the heart. Capillaries, thanks to their thin walls, allow the exchange of substances between body cells and blood. Due to their size, arteries can be divided into large (approx. 24–39 mm), which includes the aorta; medium (up to 35 mm); and small (arterioles). Their diameter is from 1.1 to 2 mm. In terms of structure, we distinguish arteries of the following types:

- Elastic (large): in their structure, elastic fibers and membranes play the main role,
- Muscular (medium and small): in their walls, there is more smooth muscle tissue.

The transition between subsequent types occurs gradually with the decreasing diameter of the vessels, as well as with the changing number of elastic fibers [176,177].

Bioengineered grafting, to be effective, requires a thoughtful selection of materials that minimize the risk of thrombogenicity and demonstrate adequate vascular activity. It is also crucial to ensure that the scaffolds stimulate the adhesion and proliferation of key cells, such as endothelial cells, vascular smooth muscle cells, and fibroblasts. The proliferation of vascular smooth muscle cells (VSMCs) on the artificial construct is of particular importance, as these cells are the basic elements of vascular tissue and play an important role in the process of vessel morphogenesis. In the context of bioengineered vascular grafts, the mechanical properties of the materials used are also important. They should be similar to those of native blood vessels to prevent mismatch at the interface with adjacent tissues, which is one of the main causes of graft failure. For scaffolds to function properly, they must have sufficient strength to withstand blood pressure and provide adequate stability for sutures, while remaining sufficiently flexible and soft, which is crucial for their integration with the surrounding tissues. In this way, the risk of complications can be minimized and the overall functionality of vascular grafts can be improved [150,178].

#### Polyurethane Scaffolds for Vascular Engineering

The development of small artificial blood vessels with an internal diameter of less than 6 mm poses a significant challenge in regenerative medicine and vascular surgery. Problems associated with their implantation, such as ease of embolization and low patency, are the result of many factors, including improper adaptation to the circulatory system and the body’s reaction to the material from which they are made. The high elasticity of PU can withstand repeated stress, mimicking the native blood vessel subjected to blood flow.

**Figure 16:**
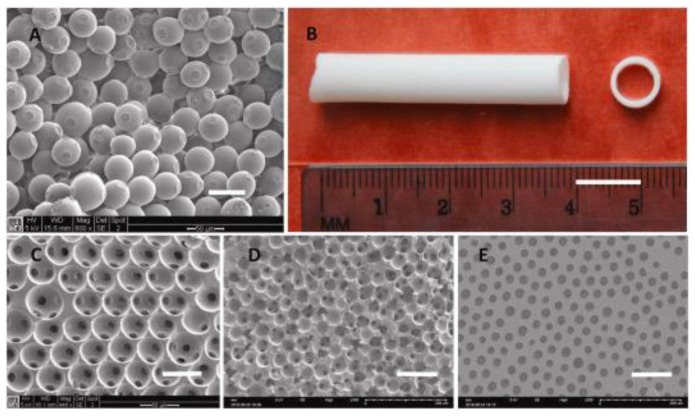
Microscopic and macroscopic structures. (**A**) An SEM image of a broken cross-section of sintered PMMA beads (small circular markings on bead surfaces were interconnects between beads). Scale bar: 50 μm. (**B**) A photograph of the side view and cross-section of a porous PU graft. Scale bar: 1 cm. (**C**) An SEM image of the exterior surface of a porous PU graft. Scale bar: 50 μm. (**D**) An SEM image of the cross-section of a porous PU graft. Scale bar: 100 μm. (**E**) An SEM image of the luminal surface of a porous PU graft., Scale bar: 100 μm. Reprinted from ref. [180], Copyright 2021, Elsevier.

The work of Nafiseh Jirofti et al. [179] suggests that optimized co-electro-coupled PCL/PU grafts can provide sufficient mechanical tension to conform to and mimic natural blood vessels. A biodegradable elastomeric polyurethane (PU) was designed as a coating for a drug-eluting stent to minimize the risk of thrombosis and to allow controlled release of an antiproliferative drug. This drug is intended to inhibit smooth muscle cell proliferation, which is crucial in preventing vasoconstriction following angioplasty procedures. Zhen et al. [180] developed biostable, cross-linked polyurethane formulations which were further used to fabricate scaffolds with precisely designed 40 μm pores. The mechanical properties of these scaffolds were matched to those of native blood vessels by optimizing the polyurethane composition. The studies showed that these scaffolds support the healing process and mitigate the body’s foreign body response (FBC). The biomaterial obtained is characterized by tunable mechanical properties and a precisely designed porous structure (Figure 16), making it a promising candidate for applications in pro-healing vascular grafts and in situ vascular engineering.

Mostafavi et al. [181] developed a novel sodium alginate-based supramolecular bioactive elastomer (BASPU) that exhibits high strength and advantageous mechanical properties. BASPU utilizes two different physical networks: the first consists of soft ammonium–soft tertiary sulfate pairs that function as strong ionic bonds, and the second consists of soft tertiary carboxylate groups that function as weak bonds. The presence of sulfate groups in the elastomer structure contributes to a low Young’s modulus, which means that the material is flexible while offering high strength and excellent tensile properties. Additionally, BASPU exhibits efficient energy dissipation, ultrafast self-healing ability, and high overall healing efficiency. In vitro studies have shown that BASPU has improved endothelial cell adhesion, which is important for integration with biological tissues. In addition, this material demonstrates higher antithrombotic capacity and significantly lower platelet adhesion compared to commercial artificial vessels.

Kianpour et al. [182] presented a novel approach to the production of scaffolds for artificial vascular grafts using a flexible, porous, and biocompatible titanium dioxide nanotube (TNT) film with polyurethane (PU). The study compared the mechanical and biological properties of open (OE) and closed (CE) TNT-PU films with their pure PU counterparts. The results of the study showed that the structure of the OE-TNT-PU scaffolds was not only hydrophilic but also superhydrophilic, which promoted better cell attachment and proliferation. It was noted that the endothelialization rate in the OE-TNT-PU scaffolds was twice as high as that of pure PU scaffolds after 5 days of in vitro culture. Moreover, these scaffolds were characterized by significantly lower platelet counts and agglomeration, which may indicate better biocompatibility of the material. The mechanical properties of the OE-TNT-PU films were considerably improved, compared to pristine PU: the elongation at break was as high as 881% and the tensile strength was more than three times higher than that of pure PU. The Young’s modulus of these scaffolds exceeded 150 MPa, suggesting that these materials are exceptionally flexible and strong, which is crucial for applications in regenerative medicine and vascular surgery.

Small-diameter vascular scaffolds developed by co-electrospinning using poly(ε-caprolactone) (PCL) and flexible polyurethane (PU) exhibit promising mechanical and biological properties [179]. In vivo studies conducted in rat and sheep models confirmed that increasing the PU content in the scaffolds significantly improves their biocompatibility, reaching 43% with a maximum PU content of 90%. Ultimate tensile strength (UTS) measurements for different PU proportions (10%, 25%, 50%, 75% and 90%) indicate variability in mechanical properties, with the lowest UTS value obtained for 75% PU (2.2 ± 0.34 MPa) and the highest for 10% PU (4.7 ± 0.34 MPa). Cytotoxicity studies conducted using the MTT assay showed that the number of cells on the scaffolds increased compared to the control group, suggesting a beneficial effect of the materials on cell proliferation from day one to day seven. The fabricated composite structures as vascular scaffolds provided appropriate mechanical and biological properties with the desired clinical performance, and the simple preparation method makes the composite structure a good candidate for the required potentials for use as small-diameter vascular grafts in vivo.

**Figure 17:**
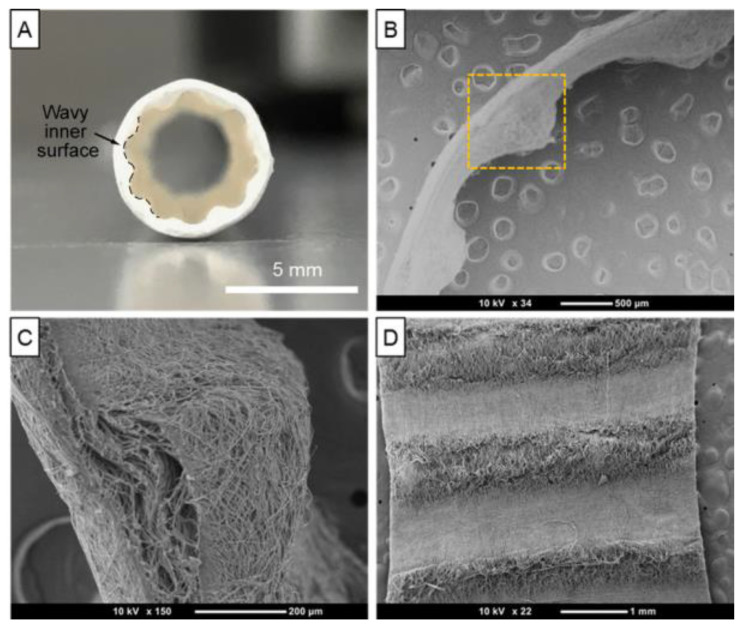
(**A**) TPU/fibroin (1:1) graft with a wavy inner surface and a smooth outer surface. (**B**) The wall of the graft was composed of a thick layer with various thicknesses and a thin skin layer. (**C**) The crest of the inner layer (the squared region in (**B**)) was formed by the accumulation of crimped fibers. (**D**) Fiber morphology of the inner surface. Reprinted from ref. [183], Copyright 2018, Wiley.

Yu et al. [183] prepared by electrospinning hybrid vascular grafts that combines the properties of synthetic thermoplastic polyurethane (TPU) and natural fibroin. The use of a rotating collector allowed obtaining a unique fibrous structure with a biomimetic architecture, which contributed to increased flexibility of the graft compared to traditional, regularly aligned fibers (Figure 17).

The results showed that the obtained fiber morphologies differed depending on the materials and processing parameters used. The TPU/fibroin hybrid grafts showed mechanical properties comparable to those of natural blood vessels. In addition, contact angle tests showed that TPU is hydrophobic, while fibroin is hydrophilic. The combination of these two materials allowed obtaining grafts with balanced hydrophilicity, which favors cellular activity and interaction with the matrix. Importantly, a significantly higher cell population was observed in the case of the TPU/fibroin hybrid graft compared to the graft made of TPU alone. This indicates that the presence of fibroin significantly promotes cell proliferation.

## 7. Advantages and Disadvantages of Polyurethanes Compared to Other Polymer Biomaterials

Polyurethanes (PU) are versatile biomaterials with unique properties that make them an attractive option for medical applications. Their flexibility and modifiability mean they can be tailored to a variety of clinical needs. Below, we discuss the advantages and disadvantages of polyurethanes compared to other medical polymers such as PCL (polycaprolactone), PLA (polylactic acid), and PLGA (poly(lactic–glycolic acid) acid) [184]. With such a wide range of polymeric biomaterials available, it is important to understand what unique properties make PUs a desirable option and what the potential disadvantages are.

Among the advantages of polyurethanes (PUs) are:

Customizability: The ability to modify chain length, degree of crystallinity, and the use of a variety of isocyanates and polyols allows PUs to have a wide range of mechanical, chemical, and biological properties. This flexibility allows the material to be tailored to the specific requirements of the application, which is crucial in biomedicine.

Elastomer properties: The structure of PU block copolymers allows for the creation of materials with elastomeric properties. This allows PUs to be used in applications such as implants that require both flexibility and strength.

Degradability: Although PUs are not as readily degradable as some other biopolymers, their structure can be engineered to allow controlled degradation. This is important in the context of medical applications, where materials must degrade over a specified period of time to assist in the healing process.

Disadvantages of polyurethanes (PUs) include:

Complexity of synthesis: The process of producing PUs is more complex than some other polymers. It requires often a two-step synthesis and the use of specific catalysts, which can be difficult to control and affect the quality of the final product.

Cost: The higher complexity of synthesis and the greater number of raw materials required for PU production lead to higher costs compared to simpler polymers such as PCL. This can be a significant factor when selecting a material for commercial use.

Potential biological issues: PUs can cause immunological reactions or other undesirable biological effects. Further research is needed to better understand these key issues and optimize the properties of PUs to make them safe for medical applications [185,186].

Comparison with other polymers:

PCL: It is more biodegradable and easier to synthesize, but does not offer the same flexibility and conformability as PU [187].

PLA: It has good mechanical properties, but is relatively stiff and is less elastic than polyurethanes. This limits its use in some applications where greater elasticity is required [188].

PLGA: It is an excellent material for drug delivery applications due to its controlled degradation, but has limited mechanical properties compared to PU [189].

Polyurethanes offer unique properties that can be beneficial in many biomedical applications. Their ability to be modified to obtain desired mechanical properties makes them a versatile material. Despite some drawbacks, such as the complexity of synthesis and potential biological problems, PUs remain an attractive option in the context of emerging medical technologies, especially in the area of implants.

## 8. Conclusions and Future Trends

Over the past decade, polyurethanes (PUs) have continued to gain recognition as versatile polymeric biomaterials in medicine; this is thanks to their unique physicochemical properties and the possibility of manipulating their structure. Polyurethanes are polymers that combine different macromeric building blocks to create materials with specific properties, such as biocompatibility, flexibility, strength, and durability. These characteristics make polyurethanes an attractive option for many biomedical applications. The use of biomimicry, self-assembly, and tailored degradation in polyurethane design allows for the creation of macromolecular systems that meet the requirements of complex processes, such as tissue reconstruction. Among synthetic biodegradable polymers, polyurethanes stand out as one of the most versatile classes, capable of producing scaffolds with a variety of architectures, pore sizes, and mechanical properties. By controlling the degradation rate and developing new technologies, scientists can design polyurethane-based scaffolds, including those based on non-isocyanate polyurethanes, with different biomimetic features, enabling their use in both soft- and hard-tissue regeneration. This opens up new perspectives in therapy and treatment, as well as in tissue engineering, where polyurethanes can be used to create implants, scaffolds, and other biomaterials supporting regenerative processes.

PU scaffolds support the growth of cells and tissues under controlled degradation conditions to obtain non-cytotoxic degradation products. Numerous long-term studies have confirmed the safety of PU in various animal models and provided evidence of their complete degradation. The development of reactive, injectable PU prepolymer systems that can be administered by minimally invasive surgical procedures has opened up new possibilities for the creation of biodegradable bone cements and fracture stabilizing products in orthopedics.

Among the various methods of PU forming, 3D printing has become a particularly popular research direction in biomedicine. This technology allows the creation of complex structures with high precision, maintaining good mechanical properties and biological compatibility. Compared to traditional methods, 3D printing is a faster process, allowing simultaneous forming without the need for multiple manual corrections, which significantly increases production efficiency. Future research will most likely deal with 3D printing optimization, as well as fabrication of hybrid materials based on PU. New research directions will probably focus on new PU composites containing bio-based additives and nanoadditives to better meet the needs of the modern biomedical field. A promising research field is non-isocyanate polyurethane-based scaffolds with improved properties and performance. However, this research line is at the infancy stage and requires much effort to establish structure–property relationships. As the quality and effectiveness of medical treatments are improved, the demand for advanced polyurethanes in biomedicine will continue to grow.

Recently, new technologies such as material design based on machine learning (ML) and artificial intelligence (AI) have attracted the attention of researchers in the biomaterials area. These new technologies have great potential, and their development can revolutionize many aspects of tissue engineering and biomaterials. As these technologies become more advanced, their applications in areas such as scaffold design, prediction of biological responses, or optimization of drug delivery are becoming more realistic and practical. Machine learning can support the development of materials that respond to changes in the environment (e.g., changes in temperature, pH). This allows the design of polyurethane scaffolds that can adjust their properties in response to changing conditions, which is especially important in biomedical applications.

A key aspect worth emphasizing is the need to use large data sets that can be analyzed by ML algorithms. This allows for obtaining accurate predictions of the behavior of biomaterials under different conditions, which in turn allows for their optimization. For example, in tissue engineering, the ability to predict how scaffolds will function in the body can lead to the creation of more effective and personalized solutions.

Interdisciplinary collaboration is essential to effectively use the potential of ML in biomaterials. Collaboration between specialists in different fields—such as materials science, computer science, biology, and ethics—will allow for the development of models that are not only effective, but also interpretable and ethically sound. The challenges we will face include not only technical aspects, but also issues related to ethics and responsibility in research.

As technology evolves, it will also be important to ensure that ML algorithms are transparent and understandable, which can help build trust in the results and their applications. A holistic approach to biomaterials development that combines advanced technologies with ethical responsibility can bring significant benefits to both science and clinical practice. Therefore, integrating machine learning into the design of polyurethane scaffolds opens up new possibilities for creating innovative, functional, and user-friendly materials.
